# Double Intramolecular Transacetalization of Polyhydroxy Acetals: Synthesis of Conformationally-Restricted 1,3-Dioxanes with Axially-Oriented Phenyl Moiety

**DOI:** 10.3390/molecules21111503

**Published:** 2016-11-09

**Authors:** Samuel Asare-Nkansah, Bernhard Wünsch

**Affiliations:** 1 Institut für Pharmazeutische und Medizinische Chemie der Westfälischen Wilhelms-Universität Münster, Corrensstraße 48, D-48149 Münster, Germany; asn12002@yahoo.com; 2 Cells-in-Motion Cluster of Excellence (EXC 1003–CiM), Westfälische Wilhelms-Universität Münster, D-48149 Münster, Germany

**Keywords:** double intramolecular transacetalization, polyhydroxy acetals, conformationally-restricted 1,3-dioxanes, axially-oriented phenyl moiety, tricyclic alcohols, 1,5-epoxy-2-benzoxepines

## Abstract

The synthesis of conformationally-restricted 1,3-dioxanes with a phenyl moiety fixed in an axial orientation at the acetalic center is described. Starting with diethyl 3-hydroxyglutarate (**15**), benzaldehyde acetal **12a** and acetophenone ketal **12b** bearing a protected 1,3,5-trihydroxypentyl side chain in the *o*-position were prepared. The first acid-catalyzed intramolecular transacetalization gave a mixture of diastereomeric 2-benzofurans **18** (ratio of diastereomers 2:2:1:1). After OH group deprotection, the second intramolecular transacetalization afforded tricyclic alcohol **14a** (2-(1,5-epoxy-1,3,4,5-tetrahydro-2-benzoxepin-3-yl]ethan-1-ol). Analogous cyclizations led to the corresponding silyl ether **22a** (19%) and azide **23a** (13%). Whereas tricyclic alcohol **14a** was obtained as a 1:1 mixture of diastereomers, the silyl ether **22a** and the azide **23a** afforded only one diastereomer. This observation indicates a faster cyclization of the minor diastereomers providing the thermodynamically-favored compounds with equatorially-oriented substituents in the 3-position of the tricyclic 1,5-epoxy-2-benzoxepine system. In general, acetophenone-derived ketalic compounds (**b**-series) required very mild reaction conditions and gave lower yields than the corresponding acetalic compounds (**a**-series).

## 1. Introduction

**Figure 1:**
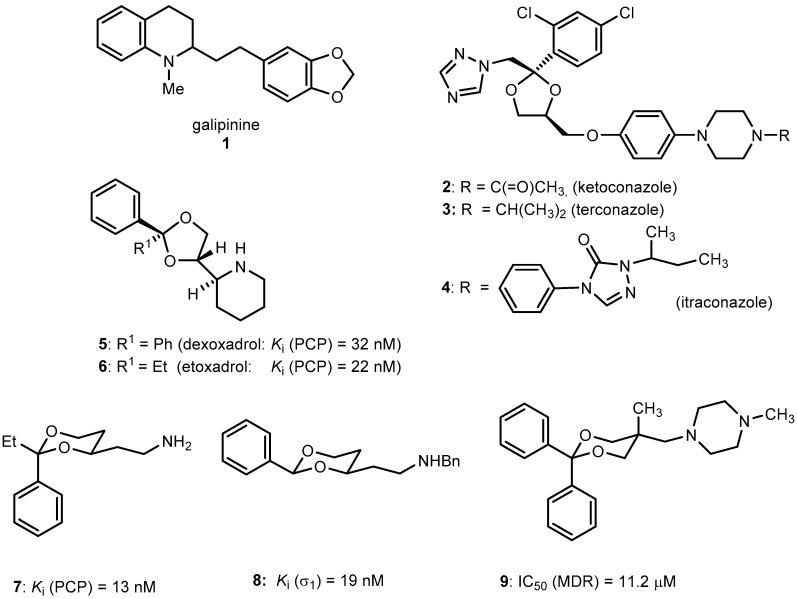
Some biologically-important compounds containing the 1,3-dioxole, 1,3-dioxolane, and 1,3-dioxane scaffold.

Heterocycles with two *O*-atoms in the acetalic position, such as 1,3-dioxoles, 1,3-dioxolanes, and homologous 1,3-dioxanes, are found in several important biologically-active compounds. The 1,3-dioxole ring is present in the natural product galipinine (**1**), found in the roots of *Galipea officinalis*, which is active against some chloroquine-resistant *Plasmodium falciparum* strains [1]. The broad-spectrum antifungal agents ketoconazole (**2**), terconazole (**3**), and itraconazole (**4**) [2,3,4], contain the 1,3-dioxolane ring. This five-membered heterocycle is also found in dexoxadrol (**5**) and etoxadrol (**6**), showing analgesic and anesthetic activity due to uncompetitive antagonism at the *N*-methyl-d-aspartate (NMDA) receptor [5,6,7] (Figure 1).

Enlargement of the 1,3-dioxolane ring of dexoxadrol (**5**) and etoxadrol (**6**) led to 1,3-dioxanes with high NMDA and σ_1_ receptor antagonistic activity. The 1,3-dioxane **7** with an aminoethyl substituent at the 4-position is a potent uncompetitive antagonist at the phencyclidine (1-(1-phenylcyclohexyl)piperidine, PCP) binding site of the NMDA receptor (*K*_i_ = 13 nM [6], Figure 1). The PCP affinity of the 1,3-dioxane **7** is even higher than the PCP affinity of the corresponding 1,3-dioxolane derivative [6]. Removal of the ethyl moiety at the acetalic position and the introduction of a benzyl moiety at the N-atom led to 1,3-dioxane **8** with very high σ_1_ receptor affinity (*K*_i_ = 19 nM). Moreover, 1,3-dioxane **8** shows very high analgesic activity in the capsaicin assay [5]. 1,3-Dioxane **9** with substituents at the 5-position in addition to two phenyl moieties at the 2-position, reveals multi-drug resistance (MDR) modulatory properties (IC_50_ = 11.2 μM) [8], which are even higher than the MDR modulatory activities of the analogous 1,3-dioxolane with a similar substitution pattern [6,8,9].

A common structural feature of the pharmacologically-active compounds **2**–**9** shown in Figure 1 is the phenyl moiety at the acetalic position. Structure-activity-relationship (SAR) studies with flexible 1,3-dioxolane- and 1,3-dioxane-based NMDA receptor antagonists led to the hypothesis that one phenyl moiety in an axially perpendicular orientation at the acetalic 2-position of the oxygen heterocycle is required for high NMDA receptor affinity [5,10,11]. 1,3-Dioxane **8** with an equatorially oriented phenyl moiety at the 2-position shows rather low PCP affinity, but rather high σ_1_ receptor affinity [5].

**Figure 2:**
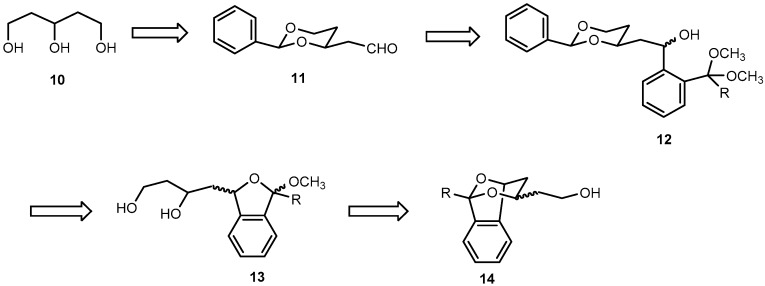
Plan for the synthesis of tricyclic compounds **14**. The phenyl ring of **14** is fixed in the axial position of the 1,3-dioxane ring without any rotational freedom.

In order to obtain insight into the preferred orientation of the phenyl moiety at the 1,3-dioxane ring, conformationally-restricted 1,3-dioxanes of type **14** (Figure 2) were envisaged bearing a phenyl moiety, which is forced to adopt an axial orientation. Herein we report on the synthesis of the tricyclic compounds **14** resulting from the 1,3-dioxanes **7**–**9** by connecting a phenyl moiety with the carbon atom at the 6-position of the 1,3-dioxane ring. In the tricyclic compounds **14** the phenyl ring is fixed in the axial orientation and, moreover, its rotation along the phenyl-C(acetal) bond is blocked.

## 2. Synthesis

**Scheme 1:**
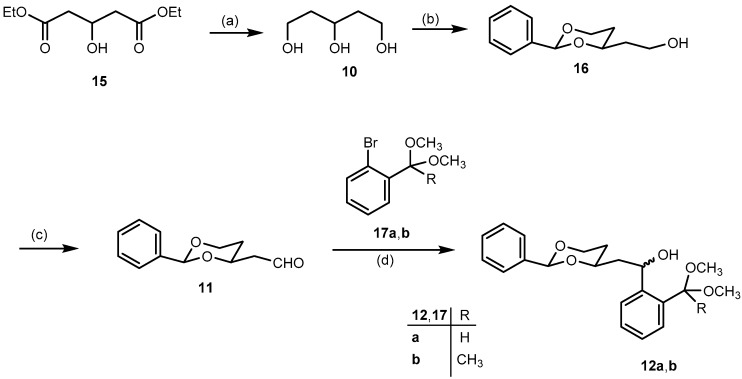
Synthesis of alcohols **12a**,**b** as key intermediates for the preparation of conformationally-restricted 1,3-dioxanes. Reagents and reaction conditions: (**a**) LiAlH_4_, THF, 66 °C, 72 h, 97%; (**b**) PhCH=O, TosOH, Na_2_SO_4_, CH_2_Cl_2_, r.t., 24 h, 85%; (**c**) (COCl)_2_, DMSO, Et_3_N, CH_2_Cl_2_, −78 °C, 92%; (**d**) *n*-BuLi, THF, −78 °C to r.t., 16 h, 93% (**12a**), 97% (**12b**).

The plan for the synthesis of tricyclic compounds **14** is based on two subsequent intramolecular transacetalization reactions. At first, aldehyde **11** will be prepared by acetalization of pentane-1,3,5-triol (**10**) with benzaldehyde and subsequent oxidation of the primary alcohol. Reaction of **11** with an arylmetal derivative bearing an acetalic substituent in the *o*-position will provide the secondary alcohols **12** (Scheme 1). Starting from **12**, the first intramolecular transacetalization will establish the benzofuran ring, and the second intramolecular transacetalization after cleavage of the 1,3-dioxane ring (**13**) will afford the tricyclic system **14**.

For the synthesis of pentane-1,3,5-triol (**10**) the diester **15** was reduced with LiAlH_4_. Careful workup resulted in 97% yield of triol **10** [12]. (Scheme 1) Acetalization of benzaldehyde with triol **10** afforded stereoselectively the *cis*-configured 1,3-dioxane **16** in 85% yield [13,14,15]. Swern oxidation [16] of the primary alcohol **16** with DMSO and oxalyl chloride provided aldehyde **11** in 92% yield [13].

Treatment of aryl bromides **17a**,**b** [17,18] with *n*-butyllithium at −78 °C led to the corresponding aryllithium intermediates, which reacted with the aldehyde **11** to give the secondary alcohols **12a**,**b**. Both alcohols **12a** and **12b** were isolated as a mixture of two diastereomers in the ratio 2:1. The ratio of diastereomeric alcohols **12a** and **12b** was determined by integration of characteristic methyne signals (PhC*H*(dioxane), R_2_C*H*OH, PhC*H*(OCH_3_)_2_) in the ^1^H-NMR spectra.

**Scheme 2:**
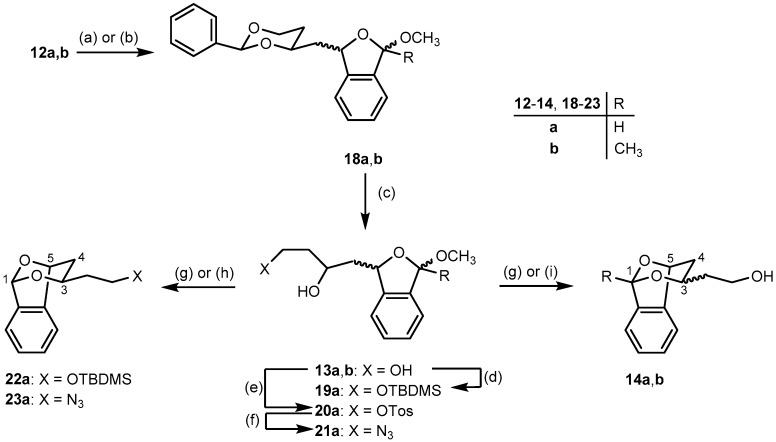
Synthesis of conformationally restricted 1,3 dioxanes **14**, **22** and **23**. Reagents and reaction conditions: (**a**) *p*TosOH, toluene, r.t., 2 h, 89% (**18a**); (**b**) *p*TosOH (0.5 mg/mL), toluene/cyclohexane (1:1), r.t., 2 h, 71% (**18b**); (**c**) *p*TosOH, MeOH/H_2_O (4:1), r.t., 36–72 h, 87% (**13a**), 55% (**13b**); (**d**) ^t^BuMe_2_SiCl, imidazole, DMF, r.t., 24 h, 58%; (**e**) *p*TosCl, Bu_2_SnO, Et_3_N, CH_2_Cl_2_, 40 °C, 48 h, 51%; (**f**) NaN_3_, DMF, 90 °C, 3 h, 92%; (**g**) *p*TosOH (0.5 mg/mL), toluene, 85 °C, 72 h, 17% (**14a**), 13% (**23a**); (**h**) 0.1 M HCl in AcOH (15 μL), toluene (5 mL), 65 °C, 60 h, 19% (**22a**); and (**i**) toluene/AcOH (4:1), r.t., 72 h, 6% (**14b**). *p*TosOH = *p*-toluenesulfonic acid.

The intramolecular transacetalization of **12a** required careful optimization of the reaction conditions in order to avoid the cleavage of the 1,3-dioxane moiety. Various acid catalysts in different solvents (e.g., *p*TosOH in CH_2_Cl_2_, AcOH in CH_2_Cl_2_, and AcOH in CHCl_3_) were investigated. The highest yield of the 2-benzofuran **18a** (89%) was obtained using *p*TosOH in toluene within 2 h at room temperature. However, the concentration of *p*TosOH has to be carefully controlled in order to avoid complete decomposition of the compounds (Scheme 2).

Unexpectedly, treatment of the alcohol **12b** with the optimized reaction conditions for the cyclization of **12a** to **18a** led to decomposition within 45 min. It is assumed that the higher reactivity of the ketal of **12b** (forming a more stable carbenium ion) compared to the acetal **12a** is responsible for this decomposition reaction. Therefore, milder reaction conditions were investigated. Low amounts of *p*TosOH in a 1:1-mixture of cyclohexane and toluene afforded the desired benzofuran **18b** in 71% yield.

For the second intramolecular transacetalization the benzaldehyde acetal of **18** protecting the hydroxy moieties of the side chain had to be cleaved. Catalytic amounts of *p*-toluenesulfonic acid (*p*TosOH) in a mixture of methanol and water (4:1) led to cleavage of the 1,3-dioxane ring, providing the diols **13a** and **13b** in 87% and 55% yields, respectively. The conversion of benzofuran **18b** with ketalic substructure into diol **13b** was slower and gave lower yields (55%) than the preparation of diol **13a** (87%) from the acetalic benzofuran **18a** (Scheme 2). In general, it was observed that substrates with a ketalic substructure (**12b**, **18b**) require milder reaction conditions than the corresponding analogues **12a** and **18a** with an acetalic system.

The benzofurans **18a**,**b**, as well as the diols **13a**,**b**, were obtained as mixtures of four diastereomers in the ratio 2:2:1:1. The ratio of diastereomers was derived from the relative intensities of the four signals for 1-H of the 2-benzofuran ring.

Although it was expected that the intramolecular transacetalization of diols **13** should easily provide six-membered acetals **14** (1,3-dioxane in the tricyclic scaffold), the synthesis of **14** was rather challenging. Several variations were investigated in order to detect the best reaction conditions. The transformation of **13a** into **14a** was performed in polar and nonpolar solvents (DMF, AcOH, CHCl_3_, toluene), with different acids (*p*TosOH, AcOH and HCl in AcOH), at r.t. up to 90 °C for periods between 3 h and 7 d producing **14a** in yields between 10%–46%. However, due to purification problems the yield of pure tricyclic alcohol **14a** (1:1 mixture of diastereomers) did not exceed 17% (*p*TosOH, toluene, 85 °C, 72 h).

As shown for the reactions of ketals **12b** and **18b**, the final cyclization starting with the ketal **13b** required very mild reaction conditions in order to avoid decomposition. Finally, the tricyclic alcohol **14b** was obtained as a 1:1 mixture of diastereomers in 6% yield after stirring the diol **13b** in a toluene/AcOH mixture at r.t. for 72 h.

It was hypothesized that the low yields of the tricyclic alcohols **14a** and **14b** could be due to competition between the secondary and primary alcohol of **13** leading to the desired compounds **14** or compounds with an eight-membered acetalic ring. Therefore, the primary alcohol of **13a** was protected regioselectively with TBDMS-Cl to provide the silyl ether **19a** in 58% yield. Treatment of the silyl ether **19a** with HCl in AcOH led to the tricyclic silyl ether **22a** in 19% yield. Although a mixture of four diastereomers (ratio 2:2:1:1) was employed in this reaction, only one diastereomer of the tricyclic silyl ether was isolated (see discussion of the stereochemistry below). The ^1^H-NMR spectrum of the recovered starting material **19a** indicated that the minor diastereomers of the silyl ether **19a** had reacted faster than the major diastereomers.

Next, the primary alcohol of **13a** should be converted into an azide. For this purpose, the diol **13a** was reacted with *p*TosCl in the presence of catalytic amounts of Bu_2_SnO [19] in refluxing CH_2_Cl_2_ to afford, regioselectively, the tosylate **20a** as a mixture of four diastereomers (ratio 2:2:1:1) in 51% yield. Substitution of the tosylate **20a** with NaN_3_ led to the azide **21a** in 92% yield (Scheme 2). It was assumed that the intramolecular transacetalization of azide **21a** should result in higher yields than the reaction of diol **13a**, since the competing primary alcohol was no longer present. The conversion of azidoalcohol **21a** into tricyclic azide **23a** was achieved with *p*TosOH in toluene (85 °C). Unexpectedly, only one isomer of **23a** was isolated in 13% yield (see discussion of the stereochemistry below). The ^1^H-NMR spectrum of the recovered starting material **21a** showed only signals for the two major diastereomers of **21a**. It was concluded that the minor diastereomers of **21a** reacted faster to form the tricyclic azide **23a** than the major diastereomers. The lower reactivity of the major diastereomers may explain the low yields of the tricyclic azide **23a**.

In order to determine the relative configuration of the diastereomerically-pure silyl ether **22a** and the azide **23a**, the ^1^H-NMR signals produced by the protons in the 3-, 4-, and 5-positions were analyzed. In both compounds the axially-oriented proton in the 4-position causes a ddd signal with two large coupling constants of 13 Hz and 11 Hz, and one small coupling constant of 3.7 Hz. The largest coupling constant (13 Hz) belongs to the geminal coupling of 4-H_ax_ with 4-H_eq_. The small coupling constant reflects the interaction between 4-H_ax_ and the equatorially-oriented 5-H. The remaining coupling constant of 11 Hz can only be explained by an 1,2-diaxial position of adjacent protons. Therefore, the proton in the 3-position has to adopt an axial orientation resulting in equatorial position of the substituted ethyl moiety in the 3-position.

## 3. Experimental Section

### 3.1. General

Unless otherwise stated, moisture-sensitive reactions were conducted under dry nitrogen. THF was dried with sodium/benzophenone and was freshly distilled before use. Similarly, dichloromethane and methanol were distilled from calcium hydride and magnesium methanolate, respectively. For the regulation of temperature below 0 °C a cryostat (EK 90, Thermo Haake, Karlsruhe, Germany) or acetone/dry ice bath (−78 °C) was used. Solvents and liquid reagents were added to the reaction mixture with disposable syringes. Thin layer chromatography (TLC): silica gel 60 F_254_ plates (Merck, Darmstadt, Germany). Flash chromatography (fc): silica gel 60, 40–64 μm (Merck); parentheses include: diameter of the column, length of column, fraction size, eluent, *R*_f_ value. IR: IR spectrophotometer IRAffinity with MIRacle 10 accessory FT-ATR-IR (Shimadzu, Duisburg, Germany). ^1^H-NMR (400 MHz), ^13^C-NMR (100 MHz): Mercury plus 400 spectrometer (Varian, Willich, Germany); ^1^H-NMR (600 MHz), ^13^C-NMR (151 MHz): Agilent Technologies (Waldbronn, Germany) 600/54 premium spectrometer: δ in ppm related to tetramethylsilane; coupling constants are given with 0.5 Hz resolution; ^1^H- and ^13^C-NMR spectra are shown in the Supplementary Materials. MS: EI = electron impact, ESI = electro spray ionization: MicroTof (Bruker Daltronics, Bremen, Germany), calibration with sodium formate clusters (ESI) and fatty acid esters (atmospheric pressure chemical ionization (APCI)) before measurement.

### 3.2. HPLC Method to Determine the Purity of the Compounds

Merck Hitachi equipment; UV detector: L-7400; autosampler: L-7200; pump: L-7100; degasser: L-7614; Method A: column: LiChrospher^®^ 60 RP-select B (5 μm), 250–4 mm cartridge; flow rate: 1.00 mL/min; injection volume: 5.0 μL; detection λ = 210 nm; solvents: A: water with 0.05% (*v*/*v*) trifluoroacetic acid; B: acetonitrile with 0.05% (*v/v*) trifluoroacetic acid: gradient elution: (A%): 0–4 min: 90%, 4–29 min: gradient from 90% to 0%, 29–31 min: 0%, 31–31.5 min: gradient from 0% to 90%, 31.5–40 min: 90%.

### 3.3. Synthetic Procedures

*Pentane-1,3,5-triol* (**10**) [12]. Under N_2_ atmosphere and ice cooling (0 °C), diethyl 3-hydroxyglutarate (**15**, 5.31 g, 26 mmol) in dry THF (10 mL) was added dropwise to a suspension of LiAlH_4_ (2.43 g, 64 mmol) in dry THF (60 mL). The reaction mixture was heated at 66 °C for 72 h. The excess of LiAlH_4_ was quenched with cold water (50 mL) under ice cooling until a completely white precipitate was observed. A solution of H_2_SO_4_ (1/4 conc.) was added dropwise to the mixture under ice cooling until the precipitate dissolved completely (pH 1). The reaction mixture was then heated to reflux for 12 h and the THF was removed under reduced pressure. The resulting aqueous mixture was alkalized with NH_3_ solution (25%) until a gelatinous precipitate of Al(OH)_3_ was observed (pH 10). The precipitate was removed by suction filtration, and the residue was washed with water (3 × 50 mL). The filtrate was concentrated in vacuo, and the residue was heated to reflux in CH_3_OH (150 mL) for 4 h. The mixture was then filtered to remove the insoluble Li_2_SO_4_. The residue was washed with CH_3_OH (3 × 50 mL). The combined filtrate was concentrated in vacuo, and the crude product was purified by fc (Ø = 6 cm, h = 15 cm, ethyl acetate:methanol = 9:1, V = 20 mL, *R*_f_ = 0.21). Colorless oil, yield 2.9 g (97%). C_5_H_12_O_3_ (120.1). Exact mass (APCI): *m*/*z* = 121.0871, calcd. 121.0859 for C_5_H_12_O_3_ [MH]^+^. ^1^H-NMR (400 MHz, CD_3_OD): δ [ppm] = 1.58–1.75 (m, 4H, C*H*_2_CHOHC*H_2_*), 3.69 (t, *J* = 6.5 Hz, 4H, C*H_2_*OH), 3.87 (tt, *J* = 8.4/4.5 Hz, 1H, C*H*OH). Signals for the OH protons are not observed in the spectrum. ^13^C-NMR (100 MHz (CD_3_)_2_SO): δ [ppm] = 40.6 (2C, *C*H_2_CHOH), 58.3 (2C, *C*H_2_OH), 65.2 (1C, *C*HOH). FTIR (neat): ν (cm^−1^) = 3298 (O-H), 2940, 2886 (C-H), 1042 (C-O).

*2-[(2RS,4SR)-2-Phenyl-1,3-dioxan-4-yl]ethanol* (**16**). *p*-Toluenesulfonic acid (50 mg, 0.3 mmol) was added to a solution of pentane-1,3,5-triol (**10**, 0.64 g, 5.3 mmol) and benzaldehyde (1.1 mL, 1.1 g, 11 mmol) in dry CH_2_Cl_2_ (25 mL). Then anhydrous Na_2_SO_4_ (3.80 g, 27 mmol) was added and the reaction mixture was stirred at r.t. for 24 h. The reaction mixture was filtered to remove Na_2_SO_4_, and the residue was washed with CH_2_Cl_2_ (3 × 10 mL). The combined filtrate was washed with saturated aqueous solution of NaHCO_3_ (3 × 10 mL). The aqueous layer was extracted with CH_2_Cl_2_ (2 × 10 mL). The combined organic layer was washed with brine (10 mL) and dried (Na_2_SO_4_). The solvent was removed in vacuo, and the crude product was purified by fc (Ø = 3 cm, h = 18 cm, cyclohexane:ethyl acetate = 1:1, V = 20 mL, *R*_f_ = 0.25). Colorless oil, yield 0.90 g (85%). C_12_H_16_O_3_ (208.3). Exact mass (APCI): *m*/*z* = 209.1155, calcd. 209.1172 for C_12_H_16_O_3_ [MH]^+^. ^1^H-NMR (400 MHz, CD_2_Cl_2_): δ [ppm] = 1.52 (dtd, *J* = 13.3/2.5/1.4 Hz, 1H, 5-*H_eq_*), 1.79 (dddd, *J* = 14.5/6.6/5.2/4.0 Hz, 1H, C*H_2_*CH_2_OH), 1.83–1.90 (m, 2H, C*H_2_*CH_2_OH (1H), 5-*H_ax_* (1H), 2.07 (t, *J* = 4.6 Hz, 1H, CH_2_O*H*), 3.73–3.84 (m, 2H, C*H_2_*OH), 3.98 (ddd, *J* = 12.4/11.4/2.6 Hz, 1H, 6-*H_ax_*), 4.10 (tdd, *J* = 11.1/4.0/2.4 Hz, 1H, 4-*H_ax_*), 4.25 (ddd, *J* = 11.4/5.1/1.4 Hz, 1H, 6-*H_eq_*), 5.53 (s, 1H, 2-*H_ax_*), 7.32–7.40 *(*m, 3H, H_arom_), 7.43–7.50 (m, 2H, H_arom_). ^13^C-NMR (100 MHz, CD_2_Cl_2_): δ [ppm] = 31.8 (1C, C-5_dioxane_), 38.9 (1C, *C*H_2_CH_2_OH), 60.4 (1C, *C*H_2_OH), 67.5 (1C, C-6_dioxane_), 76.7 (1C, C-4_dioxane_), 101.7 (1C, C-2_dioxane_), 126.6 (2C, C_arom_), 128.7 (2C, C_arom_), 129.2 (1C, C_arom_), 139.5 (1C, Cq_arom_). FTIR (neat): ν (cm^−1^) = 3410, 3383 (O-H), 2947, 2859 (C-H), 1454, 1400, 1366 (C=C_arom_), 1215, 1138, 1099 (C-O), 748, 698 (C-H_arom monosubst_).

*2-[(2RS,4RS)-2-Phenyl-1,3-dioxan-4-yl)acetaldehyde* (**11**). Under N_2_ atmosphere, a solution of oxalyl chloride in CH_2_Cl_2_ (3.0 mL, 6.0 mmol) was added in one portion to dry CH_2_Cl_2_ (10 mL) and the resulting solution was cooled down to −78 °C. Then a solution of dry (CH_3_)_2_SO (0.6 mL, 8.0 mmol) in dry CH_2_Cl_2_ (10 mL) was added dropwise (10 mL/h via a syringe pump). The reaction mixture was stirred at −78 °C for 15 min. Then a solution of alcohol **16** (0.84 g, 4.0 mmol) in dry CH_2_Cl_2_ (10 mL) was added dropwise (10 mL/h via a syringe pump), and the mixture was stirred at −78 °C for another 20 min. Finally, Et_3_N (2.9 mL, 20 mmol) was added in one portion, and the mixture was stirred at −78 °C for additional 10 min. The mixture was allowed to warm to r.t. The reaction mixture was diluted with Et_2_O (20 mL) and the precipitate was filtered off. The residue was washed with Et_2_O (2 × 20 mL) and the combined filtrate was concentrated in vacuo. The crude product was purified by fc (Ø = 3 cm, h = 17 cm, cyclohexane:ethyl acetate = 8:2, V = 20 mL, *R*_f_ = 0.30). Colorless oil, yield 0.70 g (92%). C_12_H_14_O_3_ (206.2). Exact mass (APCI): *m*/*z* = 207.1008, calcd. 207.1016 for C_12_H_15_O_3_ [MH]^+^. ^1^H-NMR (400 MHz, CD_2_Cl_2_): δ [ppm] = 1.61 (dtd, *J* = 13.3/2.5/1.4 Hz, 1H, 5-*H_eq_*), 1.86 (dddd, *J* = 13.2/12.2/11.3/5.0 Hz, 1H, 5-*H_ax_*), 2.60 (ddd, *J* = 16.8/4.8/1.6 Hz, 1H, C*H_2_*CHO), 2.74 (ddd, *J* = 16.8/7.6/2.4 Hz, 1H, C*H_2_*CHO), 3.95–4.04 (m, 1H, 6-*H_ax_*), 4.25 (ddd, *J* = 11.5/5.0/1.4 Hz, 1H, 6-*H_eq_*), 4.42 (dddd, *J* = 11.2/7.5/4.8/2.5 Hz, 1H, 4-*H_ax_*), 5.55 (s, 1H, 2-*H_ax_*), 7.30–7.41 (m, 3H, H_arom_), 7.41–7.50 (m, 2H, H_arom_), 9.81 (dd, *J* = 2.4/1.6 Hz, 1H, CH_2_C*H*O). ^13^C-NMR (100 MHz, CD_2_Cl_2_): δ [ppm] = 31.6 (1C, C-5_dioxane_), 50.0 (1C, *C*H_2_CHO), 67.3 (1C, C-6_dioxane_), 72.8 (1C, C-4_dioxane_), 101.7 (1C, C-2_dioxane_), 126.6 (2C, C_arom_), 128.6 (2C, C_arom_), 129.3 (1C, C_arom_), 139.2 (1C, Cq_arom_), 200.8 (1C, CH_2_*C*HO). FTIR (neat): ν (cm^−1^) = 2855 (C-H), 1721 (C=O), 1238, 1142, 1099 (C-O), 752, 698 (C-H_arom monosubst_).

*2-{(1RS)-2-[(2RS,4RS)-* *and* *(2SR,4SR)-4-Phenyl-1,3-dioxan-2-yl]-1-hydroxyethyl}benzaldehyde dimethyl acetal* (**12a**). Under N_2_ atmosphere, a solution of bromoacetal **17a** (0.72 g, 3.1 mmol) in dry THF (10 mL) was cooled down to −78 °C. Then 1.6 M *n*-butyllithium in *n*-hexanes (2.0 mL, 3.1 mmol) was added dropwise over 15 min, and the reaction mixture was stirred at −78 °C for 10 min. Then a solution of aldehyde **11** (0.43 g, 2.1 mmol) in dry THF (5 mL) was added dropwise over 15 min. The reaction mixture was stirred at −78 °C for 2 h, and was then allowed to warm to r.t. overnight. Under ice cooling, water (15 mL) was added dropwise to the reaction mixture. THF was removed in vacuo from the mixture and the resulting aqueous layer was extracted with CHCl_3_ (3 × 20 mL). The combined organic layer was washed with brine (15 mL), dried (Na_2_SO_4_,), filtered, and the solvent was removed in vacuo. The crude product was purified by fc (Ø = 3 cm, h = 18 cm, cyclohexane: ethyl acetate = 8:2, V = 20 mL, *R*_f_ = 0.26). Pale yellow oil, yield 0.70 g (93%), mixture of two diastereomers (2:1). C_21_H_26_O_5_ (358.4). Exact Mass (APCI): *m*/*z* = 325.1437, calcd. 325.1434 for C_20_H_21_O_4_ [M − HOCH_3_ − H]^+^; *m*/*z* = 295.1325, calcd. 295.1329 for C_19_H_19_O_3_ [M − HOCH_3_ − OCH_3_]^+^. MS (ESI): *m*/*z* = 381 [M + Na]^+^, 295 [M − HOCH_3_ − OCH_3_]^+^. ^1^H-NMR (400 MHz, CD_3_OD-*d*_4_): δ [ppm] = 1.54 (dtd, *J* = 13.3/2.6/1.4 Hz, 0.33H, 5-*Heq*^o^), 1.59 (dtd, *J* = 13.3/2.5/1.4 Hz, 0.67H, 5-*H_eq_*^m^), 1.68–1.97 (m, 2.34H, C*H_2_*CHOH^m^ (2 × 0.67H), 5-*H_ax_*^o+m^ (1H)), 2.14 (dt, *J* = 13.9/7.5 Hz, 2 × 0.33H, C*H*_2_CHOH°), 3.18 (s, 3 × 0.67H, OC*H_3_*^m^), 3.22 (s, 3 × 0.33H, OC*H_3_*^o^), 3.27 (s, 3 × 0.67H, OC*H_3_*^m^), 3.27 (s, 3 × 0.33H, OC*H_3_*^o^), 3.84–4.04 (m, 2H, 6-*H_eq_*^o^ (0.33H), 4-*H_ax_*^m^ (0.67H), 6-*H_ax_*^o+m^ (1H)), 4.12–4.28 (m, 1H, 4-*H_ax_*^o^ (0.33H), 6-*H_eq_*^m^ (0.67H)), 5.36 (dd, *J* = 7.9/5.8 Hz, 0.67H, C*H*OH^m^), 5.42–5.47 (m, 1H, C*H*OH^o^ (0.33H), (C*H*(OCH_3_)_2_^m^ (0.67H)), 5.51 (s, 0.33H, 2-*H_ax_*^o^), 5.57 (s, 0.67H, 2-*H_ax_*^m^), 5.59 (s, 0.33H, C*H*(OCH_3_)_2_^o^), 7.20–7.29 (m, 2 × 0.67H, H_arom_), 7.30–7.40 (m, 4H, H_arom_), 7.44–7.48 (m, 2H, H_arom_), 7.49–7.55 (m, 0.67H, H_arom_), 7.59 (ddd, *J* = 7.8/4.1/1.3 Hz, 1H, H_arom_). Ratio of diastereomers 67:33. ^o^ = minor isomer; ^m^ = major isomer. ^13^C-NMR (151 MHz, DMSO-*d*_6_): δ [ppm] = 30.6^m^ (0.67C, C-5_dioxane_), 31.4^o^ (0.33C, C-5_dioxane_), 45.3^m^ (0.67C, *C*H_2_CHOH), 46.2^o^ (0.33C, *C*H_2_CHOH), 53.3^m^ (2 × 0.67C, O*C*H_3_), 53.5^o^ (2 × 0.33C, O*C*H_3_), 63.5^o^ (0.33C, *C*HOH), 64.1^m^ (0.67C, *C*HOH), 66.3^o+m^ (1C, C-6_dioxane_), 73.4^o^ (0.33C, C-4_dioxane_), 74.4^m^ (0.67C, C-4_dioxane_), 100.2^o^ (0.33C, *C*H(OCH_3_)_2_), 100.3^m^ (0.67C, *C*H(OCH_3_)_2_), 100.97^m^ (0.67C, C-2_dioxane_), 101.0^o^ (0.33C, C-2_dioxane_), 125.6^m^ (0.67C, C_arom_), 125.7^o^ (0.33C, C_arom_), 126.06^m^ (0.67C, C_arom_), 126.08^m^ (2 × 0.67C, C_arom_), 126.10^o^ (2 × 0.33C, C_arom_), 126.13^o^ (0.33C, C_arom_), 126.3^o^ (0.33C, C_arom_), 126.5^m^ (0.67C, C_arom_), 127.9^m^ (0.67C, C_arom_), 128.0^m^ (2 × 0.67C, C_arom_), 128.4^o^ (0.33C, C_arom_), 128.5^o+m^ (1C, C_arom_), 128.53^m^ (0.67C, C_arom_), 133.9^o^ (0.33C, Cq_arom_), 134.1^m^ (0.67C, Cq_arom_), 139.0^m^ (0.67C, Cq_arom_), 139.2^o^ (0.33C, Cq_arom_), 144.2^m^ (0.67C, Cq_arom_), 144.8^o^ (0.33C, Cq_arom_). Ratio of diastereomers 67:33. ^o^ = minor isomer; ^m^ = major isomer. FTIR (neat): ν (cm^−1^) = 3460 (O-H), 2947 (C-H), 1454 (C=C_aromatic_), 1099 (C-O), 752, 698 (C-H_arom mono- and 1,2-disubst_).

*1-{2-[(1RS)-2-[(2RS,4RS)- and (2SR,4SR)-2-Phenyl-1,3-dioxan-4-yl]-1-hydroxyethyl]phenyl}ethanone dimethyl ketal* (**12b**). Under N_2_ atmosphere, a solution of 2′-bromoacetophenone dimethyl ketal (**17b**, 0.95 g, 3.7 mmol) in dry THF (12 mL) was cooled down to −78 °C. Then 2.5 M *n*-butyllithium in *n*-hexanes (1.5 mL, 3.7 mmol) was added dropwise over 15 min. The reaction mixture was stirred at −78 °C for 10 min. A solution of aldehyde **11** (0.51 g, 2.5 mmol) in dry THF (6 mL) was added dropwise over 18 min. The reaction mixture was stirred at −78 °C for 2 h, then it was allowed to warm to r.t. overnight. Under ice cooling, water (15 mL) was added dropwise to the reaction mixture. THF was removed in vacuo from the mixture and the resulting aqueous layer was extracted with chloroform (3 × 20 mL). The combined organic layer was washed with brine (15 mL), dried (Na_2_SO_4_), filtered, and the solvent removed in vacuo. The crude product was purified by fc (Ø = 2 cm, h = 15 cm, cyclohexane:ethyl acetate = 8:2, V = 20 mL, *R*_f_ = 0.40). Pale yellow oil, yield 0.89 g (97%), mixture of two diastereomers (2:1). C_22_H_28_O_5_ (372.5). Exact Mass (APCI): *m*/*z* = 310.1536, calcd. 310.1569 for C_20_H_22_O_3_ [M − HOCH_3_ − OCH_3_ + H]^+^. MS (ESI): *m*/*z* = 395 [M + Na]^+^. ^1^H-NMR (400 MHz, DMSO-*d*_6_): δ [ppm] = 1.46 (s, 3 × 0.33H, C*H_3_*C(OCH_3_)_2_^o^), 1.50 (s, 3 × 0.67H, C*H_3_*C(OCH_3_)_2_^m^), 1.53–1.79 (m, 3.34H, 5-*H*_eq_^o+m^ (1H), 5-*H_ax_*^o+m^ (1H), C*H_2_*CHOH^m^ (2 × 0.67H)), 1.91–2.02 (m, 2 × 0.33H, C*H_2_*CHOH^o^), 3.01 (s, 3 × 0.67H, OC*H_3_*^m^), 3.07 (2s, 6 × 0.33H, OC*H_3_*^o^), 3.10 (s, 3 × 0.67H, OC*H_3_*^m^), 3.93 (qd, *J* = 11.4/2.7 Hz, 1H, 6-*H_ax_*^o+m^), 4.05–4.25 (m, 2H, 4-*H_ax_*^o+m^ (1H), 6-*H_eq_*^o+m^ (1H)), 4.97 (2d, *J* = 4.8 Hz, 1H, CHO*H*^o+m^), 5.34 (ddd, *J* = 10.0/5.0/3.0 Hz, 0.67H, C*H*OH^m^), 5.51 (s, 1H, 2-*H_ax_*^m^ (0.67H), C*H*OH^o^ (0.33H)), 5.55 (s, 0.33H, 2-*H_ax_*^o^), 7.18 (dtd, *J* = 8.3/7.1/1.5 Hz, 2 × 0.67H, H_arom_^m^), 7.25–7.36 (m, 4H, H_arom_^o+m^), 7.36–7.41 (m, 2 × 0.67H, H_arom_^m^), 7.44 (ddd, *J* = 8.0/6.5/1.8 Hz, 2 × 0.67H, H_arom_^m^), 7.60 (td, *J* = 8.0/1.4 Hz, 1H, H_arom_^o+m^). Ratio of diastereomers 67:33. ^o^ = minor isomer; ^m^ = major isomer. ^13^C-NMR (101 MHz, DMSO-*d*_6_): δ [ppm] = 25.7^o^ (0.33C, *C*H_3_), 25.9^m^ (0.67C, *C*H_3_), 30.9^m^ (0.67C, C-5_dioxane_), 32.0^o^ (0.33C, C-5_dioxane_), 45.9^m^ (0.67C, *C*H_2_CHOH), 46.8^o^ (0.33C, *C*H_2_CHOH), 48.85^m^ (2 × 0.67C, O*C*H_3_), 48.88^o^ (2 × 0.33C, O*C*H_3_), 63.8^o^ (0.33C, *C*HOH), 64.4^m^ (0.67C, *C*HOH), 66.77^m^ (0.67C, C-6_dioxane_), 66.80^o^ (0.33C, C-6_dioxane_), 74.1^o^ (0.33C, C-4_dioxane_), 75.2^m^ (0.67C, C-4_dioxane_), 100.6^o^ (0.33C, C-2_dioxane_), 100.9^m^ (0.67C, C-2_dioxane_), 102.1^o^ (0.33C, *C*(OCH_3_)_2_), 102.3^m^ (0.67C, *C*(OCH_3_)_2_), 126.5^o^ (2 × 0.33C, C_arom_), 126.54^m^ (2 × 0.67C, C_arom_), 126.6^o^ (0.33C, C_arom_), 126.61^o^ (0.33C, C_arom_), 126.7^m^ (0.67C, C_arom_), 126.8^m^ (0.67C, C_arom_), 128.15^o^ (0.33C, C_arom_), 128.16^m^ (0.67C, C_arom_), 128.2^m^ (0.67C, C_arom_), 128.24^o^ (0.33C, C_arom_), 128.3^o^ (2 × 0.33C, C_arom_), 128.34^m^ (2 × 0.67C, C_arom_), 128.8^o^ (0.33C, C_arom_), 128.9^m^ (0.67C, C_arom_), 138.5^o^ (0.33C, Cq_arom_), 138.7^m^ (0.67C, Cq_arom_), 139.5^m^ (0.67C, Cq_arom_), 139.7^o^ (0.33C, Cq_arom_), 144.9^m^ (0.67C, Cq_arom_), 145.6^o^ (0.33C, Cq_arom_). Ratio of diastereomers 67:33. ^o^ = minor isomer; ^m^ = major isomer. FTIR (neat): ν (cm^−1^) = 3487 (OH), 2924, 2851 (C-H), 1450 (C=C_aromatic_), 1099 (C-O), 760, 698 (C-H_arom mono- and 1,2-disubst_).

*1-Methoxy-3-[(2-phenyl-1,3-dioxan-4-yl)methyl]-1,3-dihydro-2-benzofuran* (**18a**) *(four diastereomers).* A solution of *p*-toluenesulfonic acid in toluene (0.5 mg/mL, 18 mL) was added to alcohols **12a** (0.33 g, 0.9 mmol). The reaction mixture was stirred vigorously at r.t. for 2 h. Saturated aqueous solution of NaHCO_3_ (30 mL) was added and the organic layer was separated. The aqueous layer was extracted with CH_2_Cl_2_ (2 × 10 mL). The combined organic layer was washed with brine (20 mL), dried (Na_2_SO_4_), filtered, and the solvent was removed in vacuo. The crude product was purified by fc (Ø = 2 cm, h = 15 cm, cyclohexane: ethyl acetate = 9:1, V = 10 mL, R_f_ = 0.39). Colorless oil, yield 0.27 g (89%), mixture of four diastereomers (2:2:1:1). C_20_H_22_O_4_ (326.4). Exact Mass (APCI): *m*/*z* = 295.1330, calcd. 295.1329 for C_19_H_19_O_3_ [M − OCH_3_]^+^; *m*/*z* = 221.0973, calcd. 221.1172 for C_13_H_17_O_3_ [M − PhCO]^+^. MS (ESI): *m*/*z* = 349 [M + Na]^+^, 295 [M – OCH_3_]^+^, 277 [M − OCH_3_ − H_2_O]^+^, 221 [M − PhCO]^+^. ^1^H-NMR (400 MHz, DMSO-*d*_6_): δ [ppm] = 1.59–2.02 (m, 3.34H, 5-*H_eq_*^o+m^ (1H), 5-*H_ax_*^o+m^ (1H), CHC*H_2_*CH^o+m+o^ (2 × 0.67H)), 2.04–2.27 (m, 2 × 0.33H, CHC*H_2_*CH^m^), 3.33 (s, 3 × 0.17H, OC*H_3_*^o^), 3.34 (s, 3 × 0.33H, OC*H_3_*^m^), 3.40 (s, 3 × 0.17H, OC*H_3_*^o^), 3.41 (s, 3 × 0.33H, OC*H_3_*^m^), 3.95–4.08 (m, 1.34H, 6-*H_ax_*^o+m^ (1H), 4-*H_ax_*^o^ (2 × 0.17H)), 4.16–4.34 (m, 1.66H, 4-*H_ax_*^m^ (2 × 0.33H), 6-*H_eq_*^o+m^ (1H)), 5.34 (dt, *J* = 10.4/4.6 Hz, 0.50H, 3-H_bf_^o+m^), 5.43–5.50 (m, 0.50H, 3-H_bf_^o+m^), 5.58 (s, 0.33H, 2-*H_ax_*^m^), 5.61 (s, 0.33H, 2-*H_ax_*^m^), 5.66 (2s, 2 × 0.17H, 2-*H_ax_*^o^), 6.12 (s, 0.33H, 1-H_bf_^m^), 6.14 (s, 0.17H, 1-H_bf_^o^), 6.20 (d, *J* = 2.1 Hz, 0.17H, 1-H_bf_^o^), 6.22 (d, *J* = 2.1 Hz, 0.33H, 1-H_bf_^m^), 7.34–7.45 (m, 16 × 0.33H + 18 × 0.17H, H_arom_), 7.48–7.54 (m, 2 × 0.33H, H_arom_). Ratio of diastereomers 33:33:17:17. ^o^ = minor isomer; ^m^ = major isomer; _bf_ = benzofuran. ^13^C-NMR (101 MHz, DMSO-*d*_6_): δ [ppm] = 31.0^m^ (0.33C, C-5_dioxane_), 31.2^m^ (0.33C, C-5_dioxane_), 32.1^o^ (2 × 0.17C, C-5_dioxane_), 41.9^m^ (0.33C, CH*C*H_2_CH), 43.0^o^ (0.17C, CH*C*H_2_CH), 43.5^m^ (0.33C, CH*C*H_2_CH), 44.7^o^ (0.17C, CH*C*H_2_CH), 54.0^o^ (0.17C, O*C*H_3_), 54.1^m^ (0.33C, O*C*H_3_), 54.3^o^ (0.17C, O*C*H_3_), 54.5^m^ (0.33C, O*C*H_3_), 66.6^o+m^ (0.50C, C-6_dioxane_), 66.7^m^ (0.33C, C-6_dioxane_), 67.4^o^ (0.17C, C-6_dioxane_), 73.8^m^ (0.33C, C-4_dioxane_), 74.0^o^ (0.17C, C-4_dioxane_), 74.1^m^ (0.33C, C-4_dioxane_), 74.2^o^ (0.17C, C-4_dioxane_), 78.8^o^ (0.17C, C-3), 79.0^o^ (0.17C, C-3), 79.03^m^ (0.33C, C-3), 79.3^m^ (0.33C, C-3), 100.5^m^ (0.33C, C-2_dioxane_), 100.6^m^ (0.33C, C-2_dioxane_), 100.7^o^ (0.17C, C-2_dioxane_), 100.74^o^ (0.17C, C-2_dioxane_), 106.2^o^ (0.17C, C-1), 106.3^m^ (0.33C, C-1), 106.6^o^ (0.17C, C-1), 106.7^m^ (0.33C, C-1), 121.6^o^ (0.17C, C_arom_), 121.8^o^ (0.17C, C_arom_), 121.84^m^ (0.33C, C_arom_), 122.0^m^ (0.33C, C_arom_), 123.4^m^ (2 × 0.33C, C_arom_), 123.42^o^ (2 × 0.17C, C_arom_), 126.4^m^ (2 × 0.33C, C_arom_), 126.42^m^ (2 × 0.33C, C_arom_), 126.5^o^ (2 × 0.17C, C_arom_), 126.53^o^ (2 × 0.17C, C_arom_), 128.06^m^ (0.33C, C_arom_), 128.10^o+m^ (0.50C, C_arom_), 128.2^o^ (0.17C, C_arom_), 128.26^m^ ( 2 × 0.33C, C_arom_), 128.3^m^ (2 × 0.33C, C_arom_), 128.38^o^ (2 × 0.17C, C_arom_), 128.39^o^ (2 × 0.17C, C_arom_), 128.8^m^ (0.33C, C_arom_), 128.81^o^ (2 × 0.17C, C_arom_), 128.9^m^ (0.33C, C_arom_), 129.6^o+m^ (0.50C, C_arom_), 129.61^o+m^ (0.50C, C_arom_), 137.7^o^ (0.17C, Cq_arom_), 137.74^o^ (0.17C, Cq_arom_), 137.8^m^ (2 × 0.33C, Cq_arom_), 139.3^o^ (2 × 0.17C, Cq_arom_), 139.34^m^ (0.33C, Cq_arom_), 139.4^m^ (0.33C, Cq_arom_), 143.3^m^ (0.33C, Cq_arom_), 143.4^m^ (0.33C, Cq_arom_), 143.5^o^ (0.17C, Cq_arom_), 143.51^o^ (0.17C, Cq_arom_). Ratio of diastereomers 33:33:17:17. ^o^ = minor isomer; ^m^ = major isomer. FTIR (neat): ν (cm^−1^) = 2947, 2858 (C-H), 1458 (C=C_aromatic_), 1096 (C-O), 752, 698 (C-H_arom mono- and 1,2-disubst_).

*1-Methoxy-1-methyl-3-[(2-phenyl-1,3-dioxan-4-yl)methyl]-1,3-dihydro-2-benzofuran* (**18b**) (*four diastereomers*). A solution of *p*-toluenesulfonic acid in toluene (25 mL, 1 mg/mL) was added to a solution of alcohols **12b** (0.89 g, 2.4 mmol) in cyclohexane (25 mL). The reaction mixture was stirred vigorously at r.t. for 2 h. Saturated aqueous solution of NaHCO_3_ (30 mL) was added and the organic layer was separated. The aqueous layer was extracted with CH_2_Cl_2_ (2 × 10 mL). The combined organic layer was washed with brine (20 mL), dried (Na_2_SO_4_), filtered, and the solvent was removed in vacuo. The crude product was purified by fc (Ø = 2 cm, h = 15 cm, cyclohexane:ethyl acetate = 9:1, V = 10 mL, R_f_ = 0.40). Pale yellow oil, yield 0.58 g (71%), mixture of four diastereomers (2:2:1:1). C_21_H_24_O_4_ (340.4). MS (ESI): *m*/*z* = 657 [2(M − OCH3) + K]^+^, 363 [M + Na]^+^, 309 [M − OCH_3_]^+^. ^1^H-NMR (400 MHz, DMSO-*d*_6_): δ [ppm] = 1.55–1.65 (m, 3.66H, C*H_3_*C(OCH_3_)^o+m^ (3H), 5-*H_eq_*^m^ (2 × 0.33H)), 1.70–1.83 (m, 1.34H, 5-*H_eq_*^o^ (2 × 0.17H), 5-*H_ax_*^o+m^ (1H)), 1.91–2.19 (m, 2H, CHC*H_2_*CH^o+m^), 2.82 (s, 3 × 0.17H, OC*H_3_*^o^), 2.84 (s, 1.5H, OC*H_3_*^o+m^), 2.94 (s, 3 × 0.33H, OC*H_3_*^m^), 3.94 (dd, *J* = 11.7/2.9 Hz, 2 × 0.33H, 6-*H_ax_* ^m^), 3.97–4.04 (m, 0.67H, 6-*H_ax_*^o^ (2 × 0.17H), 6-*H_eq_*^m^ (0.33H)), 4.12–4.16 (m, 2 × 0.33H, 4-*H_ax_*^m^), 4.19 (ddd, *J* = 8.9/5.6/3.2 Hz, 0.33H + 2 × 0.17H, 6-*H_eq_* ^o+m+o^), 4.23 (dt, *J* = 7.1, 2.1 Hz, 2 × 0.17H, 4-*H_ax_*^o^), 5.24 (ddd, *J* = 16.6/9.6/3.5 Hz, 2 × 0.17H, 3-H_bf_^o^), 5.39–5.47 (m, 2 × 0.33H, 3-H_bf_^m^), 5.52 (s, 0.33H, 2-*H_ax_*^m^), 5.60 (s, 0.33H, 2-*H_ax_*^m^), 5.63 (2s, 2 × 0.17H, 2-*H_ax_*^o^), 7.23 (qd, *J* = 3.8/1.6 Hz, 1H, H_arom_^o+m^), 7.26–7.43 (m, 7H, H_arom_^o+m^), 7.46 (ddd, *J* = 7.4/3.8/1.9 Hz, 1H, H_arom_^o+m^). Ratio of diastereomers 33:33:17:17. ^o^ = minor isomer; ^m^ = major isomer; _bf_ = benzofuran. ^13^C-NMR (101 MHz, DMSO-*d*_6_): δ [ppm] = 26.1 ^m^ (0.33C, *C*H_3_C(OCH_3_)), 26.2^o^ (0.17C, *C*H_3_C(OCH_3_)), 26.8^m^ (0.33C, *C*H_3_C(OCH_3_)), 27.1 ^o^ (0.17C, *C*H_3_C(OCH_3_)), 30.3^m^ (0.33C, C-5_dioxane_), 30.5^o^ (2 × 0.17C, C-5_dioxane_), 31.5^m^ (0.33C, C-5_dioxane_), 41.6^m^ (0.33C, CH*C*H_2_CH), 41.8^m^ (0.33C, CH*C*H_2_CH), 42.4^o^ (0.17C, CH*C*H_2_CH), 43.2^o^ (0.17C, CH*C*H_2_CH), 48.9^m^ (0.33C, O*C*H_3_), 48.99^o^ (0.17C, O*C*H_3_), 49.2^m^ (0.33C, O*C*H_3_), 49.26^o^ (0.17C, O*C*H_3_), 66.0^o+m^ (1C, C-6_dioxane_), 73.1^m^ (0.33C, C-4_dioxane_), 73.37^o^ (0.17C, C-4_dioxane_), 73.4^o^ (0.17C, C-4_dioxane_), 73.44^m^ (0.33C, C-4_dioxane_), 76.6^m^ (0.33C, C-3), 76.66^o^ (0.17C, C-3), 78.4^o^ (0.17C, C-3), 78.7^m^ (0.33C, C-3), 99.8^m^ (0.33C, C-2_dioxane_), 100.0^o+m^ (0.50C, C-2_dioxane_), 100.1^o^ (0.17C, C-2_dioxane_), 109.8^o+m^ (0.50C, C-1), 110.1^m^ (0.33C, C-1), 110.2^o^ (0.17C, C-1), 121.0^o^ (0.17C, C_arom_), 121.2^o+m^ (0.50C, C_arom_), 121.4^m^ (0.33C, C_arom_), 121.8^o^ (0.17C, C_arom_), 121.83^o^ (0.17C, C_arom_), 121.9^m^ (2 × 0.33C, C_arom_), 125.7^m^ (2 × 0.33C, C_arom_), 125.8^m^ (2 × 0.33C, C_arom_), 125.9^o^ (2 × 0.17C, C_arom_), 126.0^o^ (2 × 0.17C, C_arom_), 127.6^m^ (2 × 0.33C, C_arom_), 127.7^m^ (2 × 0.33C, C_arom_), 127.77^m^ (4 × 0.33C, C_arom_), 127.8^o^ (0.17C, C_arom_), 127.9^o^ (0.17C, C_arom_), 128.1^m^ (0.33C, C_arom_), 128.2^m^ (0.33C, C_arom_), 128.3^o^ (0.17C, C_arom_), 128.31^o^ (0.17C, C_arom_), 128.8^o+m+m^ (2 × 0.33C + 0.17C, C_arom_), 128.9^o^ (0.17C, C_arom_), 138.6^m^ (2 × 0.33C, Cq_arom_), 138.7^o^ (2 × 0.17C, Cq_arom_), 138.72^o^ (0.17C, Cq_arom_), 138.8^m^ (0.33C, Cq_arom_), 139.0^o^ (0.17C, Cq_arom_), 139.08^m^ (0.33C, Cq_arom_), 142.3^m^ (0.33C, Cq_arom_), 142.4^o^ (0.17C, Cq_arom_), 142.7^m^ (0.33C, Cq_arom_), 142.8^o^ (0.17C, Cq_arom_). Ratio of diastereomers 33:33:17:17. ^o^ = minor isomer; ^m^ = major isomer. FTIR (neat): ν (cm^−1^) = 2932, 2855 (C-H), 1454 (C=C_arom_), 1099 (C-O), 748, 698 (C-H_arom mono- and 1,2-disubst_).

*4-(3-Methoxy-1,3-dihydro-2-benzofuran-1-yl)butane-1,3-diol* (**13a**) *(four diastereomers)*. A solution of 2-benzofuran **18a** (0.32 g, 1.3 mmol) in a mixture of methanol and water (20 mL, 4:1) was treated with *p*-toluenesulfonic acid (0.17 g, 1.0 mmol). The reaction mixture was stirred at r.t. for 48 h. A saturated aqueous solution of NaHCO_3_ (10 mL) was added_._ Methanol was evaporated in vacuo and the resulting aqueous layer was extracted with CH_2_Cl_2_ (3 × 10 mL). The combined organic layer was washed with brine (10 mL), dried (Na_2_SO_4_), filtered, and the solvent was removed in vacuo. The crude product was purified by fc (Ø = 2 cm, h = 13 cm, ethyl acetate (100%), V = 10 mL, *R*_f_ = 0.44). Pale yellow oil, yield 0.27 g (87%), mixture of four diastereomers (2:2:1:1). C_13_H_18_O_4_ (238.3). Exact Mass (APCI): *m*/*z* = 413.2047, calcd. 413.1959 for C_24_H_30_O_6_ [2(M − HOCH3) + H]^+^; *m*/*z* = 207.1090, calcd. 207.1016 for C_12_H_15_O_3_ [M − OCH_3_]^+^. MS (ESI): *m*/*z* = 467 [M + C_12_H_14_O_3_ + Na]^+^, 261 [M + Na]^+^, 207 [M − OCH_3_]^+^, 189 [M − H_2_O − OCH_3_]^+^. ^1^H-NMR (400 MHz, DMSO-*d*_6_): δ [ppm] = 1.33–1.98 (m, 4H, C*H_2_*CH_2_OH^o+m^ (2H), C*H_2_*CHOH^o+m^ (2H)), 3.24 (s, 3 × 0.33H, OC*H_3_*^m^), 3.28 (s, 3 × 0.17H, OC*H_3_*^o^), 3.27 (s, 3 × 0.17H, OC*H_3_*^o^), 3.31 (s, 3 × 0.33H, OC*H_3_*^m^), 3.46–3.57 (m, 2H, C*H_2_*OH^o+m^), 3.84–3.97 (m, 1H, C*H*OH^o+m^), 4.29–4.38 (m, 1H, CH_2_O*H*^o+m^), 4.48 (d, *J* = 5.1 Hz, 0.33H, CHO*H*^m^), 4.52 (d, *J* = 5.1 Hz, 0.33H, CHO*H*^m^), 4.60 (d, *J* = 2.5 Hz, 0.17H, CHO*H*^o^), 4.62 (d, *J* = 2.6 Hz, 0.17H, CHO*H*^o^), 5.22 (dd, *J* = 7.4/6.2 Hz, 0.33H, 1-H_bf_^m^), 5.27 (dd, *J* = 10.4/2.5 Hz, 0.17H, 1-H_bf_^o^), 5.34 (ddd, *J* = 7.4/5.1/2.0 Hz, 0.33H, 1-H_bf_^m^), 5.40 (dt, *J* = 10.2/2.3 Hz, 0.17H, 1-H_bf_^o^), 6.01 (s, 0.33H, 3-H_bf_^m^), 6.03 (s, 0.17H, 3-H_bf_^o^), 6.09 (2s, Hz, 0.50H, 3-H_bf_^o+m^), 7.24–7.41 (m, 4H, H_arom_). Ratio of diastereomers 33:33:17:17. ^o^ = minor isomer; ^m^ = major isomer; _bf_ = benzofuran. ^13^C-NMR (101 MHz, DMSO-*d*_6_): δ [ppm] = 39.8^m^ (2 × 0.33C, *C*H_2_CH_2_OH), 41.1^o^ (0.17C, *C*H_2_CH_2_OH), 41.14^o^ (0.17C, *C*H_2_CH_2_OH), 44.0^m^ (0.33C, *C*H_2_CHOH), 44.7^o^ (0.17C, *C*H_2_CHOH), 45.5^m^ (0.33C, *C*H_2_CHOH), 46.3^o^ (0.17C, *C*H_2_CHOH), 53.5^m^ (0.33C, O*C*H_3_), 53.7^o^ (2 × 0.17C, O*C*H_3_), 53.9^m^ (0.33C, O*C*H_3_), 58.0^o^ (0.17C, *C*H_2_OH), 58.05^o+m^ (0.50C, *C*H_2_OH), 58.06^m^ (0.33C, *C*H_2_OH), 64.3^o^ (0.17C, *C*HOH), 64.7^o^ (0.17C, *C*HOH), 64.72^m^ (0.33C, *C*HOH), 64.8^m^ (0.33C, *C*HOH), 79.2^o^ (0.17C, C-1), 79.6^o^ (0.17C, C-1), 79.7^m^ (0.33C, C-1), 80.0^m^ (0.33C, C-1), 105.6^m^ (0.33C, C-3), 105.7^o^ (0.17C, C-3), 106.0^o^ (0.17C, C-3), 106.1^m^ (0.33C, C-3), 121.0^o^ (0.17C, C_arom_), 121.2^o^ (0.17C, C_arom_), 121.3^m^ (0.33C, C_arom_), 121.6^m^ (0.33C, C_arom_), 122.9^m^ (2 × 0.33C, C_arom_), 123.0^o^ (2 × 0.17C, C_arom_), 127.5^o^ (0.17C, C_arom_), 127.55^m^ (0.33C, C_arom_), 127.59^o^ (0.17C, C_arom_), 127.6^m^ (0.33C, C_arom_), 129.1^m^ (0.33C, C_arom_), 129.13^o^ (0.17C, C_arom_), 129.14^m^ (0.33C, C_arom_), 129.2^o^ (0.17C, C_arom_), 137.3^o^ (0.17C, Cq_arom_), 137.4^m^ (0.33C, Cq_arom_), 137.42^m^ (0.33C, Cq_arom_), 137.5^o^ (0.17C, Cq_arom_), 143.46^m^ (0.33C, Cq_arom_), 143.50^m^ (0.33C, Cq_arom_), 143.7^o^ (0.17C, Cq_arom_), 143.8^o^ (0.17C, Cq_arom_). Ratio of diastereomers 33:33:17:17. ^o^ = minor isomer; ^m^ = major isomer. FTIR (neat): v (cm^−1^) = 3375 (O-H), 2936, 2889 (C-H), 1431 (C=C_aromatic_), 1080 (C-O), 752 (C-H_arom 1,2-disubst_).

*4-(3-Methoxy-3-methyl-1,3-dihydro-2-benzofuran-1-yl)butane-1,3-diol* (**13b**) *(four diastereomers).* A solution of 2-benzofuran **18b** (0.22 g, 0.6 mmol) in a mixture of methanol and water (12 mL, 4:1) was treated with *p*-toluenesulfonic acid (40.0 mg, 0.2 mmol). The reaction mixture was stirred at r.t. for 72 h. Saturated aqueous solution of NaHCO_3_ (10 mL) was added_._ Methanol was removed in vacuo and the resulting aqueous layer was extracted with CH_2_Cl_2_ (3 × 10 mL). The combined organic layer was washed with brine (10 mL), dried (Na_2_SO_4_), filtered, and the solvent was removed in vacuo. The crude product was purified by fc (Ø = 2 cm, h = 10 cm, ethyl acetate (100%), V = 10 mL, *R*_f_ = 0.40). Yellow oil, yield 88.4 mg (55%), mixture of four diastereomers (2:2:1:1). C_14_H_20_O_4_ (252.31). MS (ESI): *m*/*z* = 463 [2(M − HOCH3) +Na]^+^, 275 [M + Na]^+^, 203 [M − OCH_3_ − H_2_O]^+^, 221 [M − OCH_3_]^+^, ^1^H-NMR (400 MHz, CD_3_OD): δ [ppm] = 1.55–2.07 (m, 7H, C*H_3_*^o+m^ (3H), C*H_2_*CHOHC*H_2_*^o+m^ (4H)), 2.94 (s, 3 × 0.33H*, OC*H_3_*^m^), 2.95 (s, 3 × 0.17H*, OC*H_3_*^o^), 3.01 (s, 3 × 0.17H*, OC*H_3_*^o^), 3.04 (s, 3 × 0.33H*, OC*H_3_*^m^), 3.41–3.62 (m, 0.33H, CH_2_C*H_2_*OH^m^), 3.65–3.87 (m, 3 × 0.33H + 4 × 0.17H, CH_2_C*H_2_*OH^o+m^), 3.95–4.25 (m, 1H*, CH_2_C*H*OH^o+m^), 5.17–5.36 (m, 0.50H, 1-H_bf_^o+m^), 5.38–5.48 (m, 0.33H, 1-H_bf_^m^), 5.53 (dd, *J* = 10.2/2.3 Hz, 0.17H, 1-H_bf_^o^), 7.20–7.49 (m, 4H, H_arom_^o+m^). Ratio of diastereomers 33:33:17:17. Signals of the OH protons are not observed in the spectrum. * The integrals of these signals are slightly lower than expected. ^o^ = minor isomer; ^m^ = major isomer; _bf_ = benzofuran. ^13^C-NMR (101 MHz, CD_3_OD): δ [ppm] = 26.7^o^ (0.17C, C*C*H_3_(OCH_3_)), 26.73^m^ (0.33C, C*C*H_3_(OCH_3_)), 27.7^m^ (0.33C, C*C*H_3_(OCH_3_)), 27.8^o^ (0.17C, C*C*H_3_(OCH_3_)), 40.2^m^ (0.33C, *C*H_2_CH_2_OH), 40.4^m^ (0.33C, *C*H_2_CH_2_OH), 41.6^o^ (0.17C, *C*H_2_CH_2_OH), 41.63^o^ (0.17C, *C*H_2_CH_2_OH), 45.0^m^ (0.33C, *C*H_2_CHOH), 45.6^o^ (0.17C, *C*H_2_CHOH), 45.8^m^ (0.33C, *C*H_2_CHOH), 46.6^o^ (0.17C, *C*H_2_CHOH), 50.1^m^ (2 × 0.33C, O*C*H_3_), 50.5^o^ (0.17C, O*C*H_3_), 50.6^o^ (0.17C, O*C*H_3_), 60.2^o+m^ (1C, CH_2_*C*H_2_OH), 67.0^o^ (2 × 0.17C, CH_2_*C*HOH), 68.0^m^ (0.33C, CH_2_*C*HOH), 68.1^m^ (0.33C, CH_2_*C*HOH), 80.6^m^ (0.33C, C-1), 81.4^o^ (2 × 0.17C, C-1), 82.4^m^ (0.33C, C-1), 111.9^m^ (0.33C, C-3), 112.1^m^ (0.33C, C-3), 112.5^o^ (2 × 0.17C, C-3), 122.0^m^ (0.33C, C_arom_), 122.3^o^ (0.17C, C_arom_), 122.34^m^ (0.33C, C_arom_), 122.5^o^ (0.17C, C_arom_), 123.26^o^ (0.17C, C_arom_), 123.3^m^ (0.33C, C_arom_), 123.4^o^ (0.17C, C_arom_), 123.42^m^ (0.33C, C_arom_), 129.1^o^ (0.17C, C_arom_), 129.2^m^ (0.33C, C_arom_), 129.3^o^ (0.17C, C_arom_), 129.33^m^ (0.33C, C_arom_), 130.3^o^ (0.17C, C_arom_), 130.36^o^ (0.17C, C_arom_), 130.4^m^ (2 × 0.33C, C_arom_), 140.1^o^ (0.17C, Cq_arom_), 140.2^o^ (0.17C, Cq_arom_), 140.4^m^ (0.33C, Cq_arom_), 140.6^m^ (0.33C, Cq_arom_), 144.2^o^ (0.17C, Cq_arom_), 144.6^m^ (2 × 0.33C, Cq_arom_), 144.9^o^ (0.17C, Cq_arom_). Ratio of diastereomers 33:33:17:17. ^o^ = minor isomer; ^m^ = major isomer. FTIR (neat): ν (cm^−1^) = 3395 (O-H), 2935, 2881 (C-H), 1431, (C=C_aromatic_), 1041 (C-O), 764 (C-H_arom 1,2-disubst_).

*[(tert-Butyldimethylsilyl)oxy]-1-(3-methoxy-1,3-dihydro-2-benzofuran-1-yl)butan-2-ol (**19a**)* (*four diastereomers).* In DMF (0.50 mL), diols **13a** (34.1 mg, 0.14 mmol) were treated with imidazole (29.2 mg, 0.43 mmol) and tert-butyldimethylsilyl chloride (22.2 mg, 0.14 mmol). The reaction mixture was stirred at r.t. for 24 h, diluted with EtOAc (10 mL) and washed with water (4 × 5 mL). The organic phase was separated, dried (Na_2_SO_4_), filtered and was concentrated under reduced pressure. The crude product was purified by fc (Ø = 2 cm, h = 10 cm, cyclohexane: ethyl acetate (8:2), V = 10 mL, R_f_ = 0.44). Colorless oil, yield 26.6 mg (58%). C_19_H_32_O_4_Si (352.5). Ratio of diastereomers 33:33:17:17. ^1^H-NMR (400 MHz, DMSO-*d*_6_): δ [ppm] = 0.02–0.04 (m, 6H, (C*H_3_*)_2_^t^BuSi^o+m^), 0.86–0.87 (m, 9H, Me_2_ (C(C*H_3_*)_3_Si^o+m^), 1.43–1.95 (m, 4H, C*H_2_*CHOHC*H_2_*^o+m^), 3.28 (s, 3 × 0.33H, OCH_3_^m^), 3.29 (s, 3 × 0.17H*, OCH_3_^o^), 3.32 (s, 3 × 0.17H *, OCH_3_^o^), 3.34 (s, 3 × 0.33H, OCH_3_^m^), 3.68–3.78 (m, 2H, C*H_2_*OH^o+m^), 3.90–3.96 (m, 1H, C*H*OH^o+m^), 4.51 (d, *J* = 5.3 Hz, 0.33H, CHO*H*^m^), 4.53 (d, *J* = 5.3 Hz, 0.33H, CHO*H*^m^), 4.63 (d, *J* = 3.3 Hz, 0.17H*, CHO*H*^o^), 4.64 (d, *J* = 3.2 Hz, 0.17H *, CHO*H*^o^), 5.26 (dd, *J* = 7.4/6.4 Hz, 0.33H, 1-H_bf_^m^), 5.31 (dd, *J* = 10.4/2.4 Hz, 0.17H, 1-H_bf_^o^), 5.34–5.39 (dd, *J* = 10.4/3.7 Hz, 0.33H, 1-H_bf_^m^), 5.43 (dd, *J* = 11.4/1.4 Hz, 0.17H, 1-H_bf_^o^), 6.04 (s, 0.33H, 3-H_bf_^m^), 6.06 (s, 0.17H, 3-H_bf_^o^), 6.12 (2s, 0.5H, 3-H_bf_^o+m^), 7.28–7.44 (m, 4H, H_arom_). Ratio of diastereomers 33:33:17:17. * Signals were slightly lower than expected. ^o^ = minor isomer, ^m^ = major isomer, _bf_ = benzofuran.

*[3-Hydroxy-4-(3-methoxy-1,3-dihydro-2-benzofuran-1-yl)butyl-] 4-methylbenzenesulfonate* (**20a**) *(four diastereomers).* A solution of diols **13a** (0.20 g, 0.84 mmol) in dry CH_2_Cl_2_ (4 mL) was treated with Bu_2_SnO (10.7 mg, 0.04 mmol), Et_3_N (176 μL, 1.3 mmol) and *p*-toluenesulfonyl chloride (0.18 g, 0.9 mmol). The reaction mixture was heated to reflux for 16 h. The solvent was removed under reduced pressure at 30 °C. The crude product was purified by fc (Ø = 2 cm, h = 12 cm, cyclohexane: ethyl acetate (7:3), V = 10 mL, *R*_f_ = 0.36 (1st spot); 0.19 (2nd spot)). Colorless oil, yield 0.16 g (51%), mixture of four diastereomers (2:2:1:1). C_20_H_24_O_6_S (392.5). MS (ESI): *m*/*z* = 361 [M − OCH_3_]^+^, 343 [M − H_2_O − OCH_3_]^+^, 171 [M − C_13_H_17_O_3_]^+^. ^1^H-NMR (400 MHz, DMSO-*d*_6_): δ [ppm] = 1.41–2.21 (m, 4H, C*H_2_*CH_2_OSO_2_PhCH_3_^o+m^ (2H), C*H_2_*CHOH^o+m^ (2H)), 2.41 (2s, 3H^o+m^, C*H_3_*Ph), 3.26 (s, 3 × 0.33H, OC*H_3_*^m^), 3.28 (s, 3 × 0.17H*, OC*H_3_*^o^), 3.30 (s, 3 × 0.33H, OC*H_3_*^o^), 3.68–3.77 (m, 2 × 0.17H, CH_2_C*H*OH^o^), 3.81 (dt, *J* = 10.2/5.5 Hz, 2 × 0.33H, CH_2_C*H*OH^m^), 3.88–4.19 (m, 2H CH_2_C*H_2_*OSO_2_PhCH_3_^o+m^), 4.70–4.82 (m, 2 × 0.33H, CH_2_CHO*H*^m^), 4.87–5.01 (m, 2 × 0.17H, CH_2_CHO*H*^o^), 5.18 (dd, *J* = 8.1/5.3 Hz, 0.33H, 1-H_bf_^m^), 5.22–5.27 (m, 0.17H, 1-H_bf_^o^), 5.29 (ddd, *J* = 7.3/4.6/1.9 Hz, 0.33H, 1-H_bf_^m^), 5.33–5.40 (m, 0.17H, 1-H_bf_^o^), 6.03 (s, 0.33H, 3-H_bf_^m^), 6.05 (s, 0.17H, 3-H_bf_^o^), 6.08 (d, *J* = 2.0 Hz, 0.33H, 3-H_bf_^m^), 6.10 (d, *J* = 2.0 Hz, 0.17H, 3-H_bf_^o^), 7.24–7.31 (m, 1H, H_arom_^o+m^), 7.31–7.42 (m, 10 × 0.33H, H_arom_^o+m^), 7.47 (dd, *J* = 8.3/3.1 Hz, 2H, H_arom_^o+m^), 7.78 (dt, *J* = 8.3/2.1 Hz, 5 × 0.33H, H_arom_^o+m^). Ratio of diastereomers 33:33:17:17. * A signal (3 × 0.17H) for the OC*H_3_* moiety of the fourth isomer cannot be observed in the spectrum, because the water peak of DMSO-*d*_6_ is overlapping with this signal. ^o^ = minor isomer; ^m^ = major isomer; _bf_ = benzofuran. ^13^C-NMR (151 MHz, DMSO-*d*_6_): δ [ppm] = 28.7^o^ (2 × 0.17C, *C*H_3_Ph), 29.6^m^ (0.33C, *C*H_3_Ph), 30.9^m^ (0.33C, *C*H_3_Ph), 35.2^m^ (2 × 0.33C, *C*H_2_CH_2_OSO_2_PhCH_3_), 35.3^o^ (2 × 0.17C, *C*H_2_CH_2_OSO_2_PhCH_3_), 43.1^m^ (0.33C, *C*H_2_CHOH), 43.9^o^ (0.17C, *C*H_2_CHOH), 44.7^m^ (0.33C, *C*H_2_CHOH), 45.6^o^ (0.17C, *C*H_2_CHOH), 53.2^m^ (0.33C, O*C*H_3_), 53.7^m^ (0.33C, O*C*H_3_), 54.6^o^ (2 × 0.17C, O*C*H_3_), 63.2^o^ (2 × 0.17C, CH_2_*C*HOH), 63.3^m^ (2 × 0.33C, CH_2_*C*HOH), 68.1^o^ (2 × 0.17C, CH_2_*C*H_2_OSO_2_PhCH_3_), 68.2^m^ (2 × 0.33C, CH_2_*C*H_2_OSO_2_PhCH_3_), 79.0^o^ (2 × 0.17C, C-1), 79.2^m^ (2 × 0.33C, C-1), 105.4^m^ (0.33C, C-3), 105.5^o^ (0.17C, C-3), 105.8^o^ (0.17C, C-3), 105.9^m^ (0.33C, C-3), 120.9^o+m^ (0.50C, C_arom_), 121.2^o+m^ (0.50C, C_arom_), 122.7^o+m+o^ (0.33C + 2 × 0.17C, C_arom_), 122.71^m^ (2 × 0.33C, C_arom_), 127.3^o+m^ (2C, C_arom_), 127.33^o+m^ (0.50C, C_arom_), 127.4^o+m^ (0.50C, C_arom_), 128.8^o+m^ (0.50C, C_arom_), 128.9^o+m^ (0.50C, C_arom_), 129.9^o+m^ (2C, C_arom_), 132.2^m^ (0.33C, Cq_arom_), 137.0^o+m^ (0.50C, Cq_arom_), 137.06^o^ (0.17C, Cq_arom_), 142.9^m^ (0.33C, Cq_arom_), 142.92^m^ (0.33C, Cq_arom_), 143.0^o^ (0.17C, Cq_arom_), 143.2^o^ (0.17C, Cq_arom_), 144.5^o+m^ (1C, Cq_arom_). Ratio of diastereomers 33:33:17:17. ^o^ = minor isomer; ^m^ = major isomer. FTIR (neat): ν (cm^−1^) = 3506 (O-H), 2920 (C-H), 1173 (RO-SO_2_R^1^), 1096(C-O), 752, 737, 664 (C-H_arom1,2- and 1,4-disubst_).

*4-Azido-1-(3-methoxy-1,3-dihydro-2-benzofuran-1-yl)butan-2-ol* (**21a**) *(four diastereomers)*. A solution of tosylates **20a** (46.6 mg, 0.12 mmol) in dry DMF (1 mL) was treated with NaN_3_ (23.2 mg, 0.36 mmol). The reaction mixture was heated to 90 ^o^C for 3 h. The solvent was removed in vacuo. The crude product was purified by fc (Ø = 2 cm, h = 12 cm, cyclohexane: ethyl acetate (7:3), V = 10 mL, *R*_f_ = 0.48). Pale yellow oil, yield 28.7 mg (92%), mixture of four diastereomers (2:2:1:1). C_13_H_17_N_3_O_3_ (263.3). Exact Mass (APCI): *m*/*z* = 234.1121, calcd. 234.1125 for C_13_H_16_NO_3_ [M – N_2_ – H]^+^; *m*/*z* = 204.1027, calcd. 204.1019 for C_12_H_14_NO_2_ [M – N_2_ – OCH_3_]^+^. ^1^H-NMR (400 MHz, CDCl_3_): δ [ppm] = 1.64–2.16 (m, 4H, C*H_2_*CHOHC*H_2_*^o+m^), 3.26–3.74 (m, 5H, OC*H_3_*^o+m^ (3H), CH_2_C*H_2_*N_3_^o+m^ (2H)), 3.87–4.06 (m, 2 × 0.17H, CH_2_C*H*OH^o^), 4.20 (dddd, *J* = 9.5/6.9/5.1/2.2 Hz, 2 × 0.33H, CH_2_C*H*OH^m^), 5.35–5.42 (m, 0.33H, 1-H_bf_^m^), 5.46 (dd, *J* = 7.8/3.3 Hz, 0.17H, 1-H_bf_^o^), 5.54 (dt, *J* = 10.4/2.5 Hz, 0.33H, 1-H_bf_^m^), 5.63 (d, *J* = 7.6 Hz, 0.17H, 1-H_bf_^o^), 6.11 (s, 0.17H, 3-H_bf_^o^), 6.16 (s, 0.33H, 3-H_bf_^m^), 6.18 (d, *J* = 2.0 Hz, 0.17H, 3-H_bf_^o^), 6.20 (d, *J* = 2.1 Hz, 0.33H, 3-H_bf_^m^), 7.17–7.22 (m, 1H, H_arom_), 7.35–7.42 (m, 3H, H_arom_). Ratio of diastereomers 33:33:17:17. Signals due to the OH protons are not observed in the spectrum. ^o^ = minor isomer; ^m^ = major isomer; _bf_ = benzofuran. ^13^C-NMR (101 MHz, CDCl_3_): δ [ppm] = 36.7^o^ (0.17C, *C*H_2_CH_2_N_3_), 36.8^o^ (0.17C, *C*H_2_CH_2_N_3_), 36.84^m^ (0.33C, *C*H_2_CH_2_N_3_), 36.9^m^ (0.33C, *C*H_2_CH_2_N_3_), 42.4^o^ (0.17C, *C*H_2_CHOH), 43.4^m^ (0.33C, *C*H_2_CHOH), 44.1^o^ (0.17C, *C*H_2_CHOH), 44.6^m^ (0.33C, *C*H_2_CHOH), 48.5^m^ (0.33C, CH_2_*C*H_2_N_3_), 48.6^m^ (0.33C, CH_2_*C*H_2_N_3_), 48.8^o^ (2 × 0.17C, CH_2_*C*H_2_N_3_), 55.1^o+m^ (0.50C, O*C*H_3_), 55.7^m^ (0.33C, O*C*H_3_), 56.1^o^ (0.17C, O*C*H_3_), 66.5^o^ (0.17C, CH_2_*C*HOH), 66.7^o^ (0.17C, CH_2_*C*HOH), 68.8^m^ (0.33C, CH_2_*C*HOH), 69.0^m^ (0.33C, CH_2_*C*HOH), 81.1^o^ (0.17C, C-1), 81.6^o^ (0.17C, C-1), 83.8^m^ (0.33C, C-1), 84.0^m^ (0.33C, C-1), 107.1^o^ (0.17C, C-3), 107.3^m^ (0.33C, C-3), 108.2^o+m^ (0.50C, C-3), 121.27^m^ (0.33C, C_arom_), 121.30^o^ (0.17C, C_arom_), 121.5^m^ (0.33C, C_arom_), 121.53^o^ (0.17C, C_arom_), 123.6^o^ (0.17C, C_arom_), 123.62^m^ (0.33C, C_arom_), 123.64^m^ (0.33C, C_arom_), 123.7^o^ (0.17C, C_arom_), 128.6^o^ (0.17C, C_arom_), 128.7^o+m^ (0.50C, C_arom_), 128.8^m^ (0.33C, C_arom_), 130.0^o+m^ (0.50C, C_arom_), 130.04^o+m^ (0.50C, C_arom_), 137.2^m^ (0.33C, Cq_arom_), 137.3^m^ (0.33C, Cq_arom_), 137.9^o^ (0.17C, Cq_arom_), 138.0^o^ (0.17C, Cq_arom_), 142.5^o^ (0.17C, Cq_arom_), 142.7^o^ (0.17C, Cq_arom_), 142.8^m^ (0.33C, Cq_arom_), 142.9^m^ (0.33C, Cq_arom_). Ratio of diastereomers 33:33:17:17. ^o^ = minor isomer; ^m^ = major isomer. FTIR (neat): ν (cm^−1^) = 3464 (O-H), 2932 (C-H), 2091 (N=N=N), 1084 (C-O), 752 (C-H_arom 1,2-disubst_).

*2-[(1RS,3RS,5RS)- and (1RS,3SR, 5RS)-1,5-Epoxy-1,3,4,5-tetrahydro-2-benzoxepin-3-yl]ethan-1-ol* (**14a**). A solution of *p*-toluenesulfonic acid in toluene (4 mL, 0.5mg/mL) was added to diols **13a** (49.2 mg, 0.21 mmol). The reaction mixture was heated to 85 °C for 72 h. Saturated aqueous solution of NaHCO_3_ (10 mL) was added and the organic layer was separated. The aqueous layer was extracted with CH_2_Cl_2_ (2 × 10 mL). The combined organic layer was dried (Na_2_SO_4_), filtered, and the solvent was removed in vacuo. The crude product was purified by fc (Ø = 2 cm, h = 12 cm, CH_2_Cl_2_:ethyl acetate (6:4), V = 10 mL, *R*_f_ =0.46 (1st isomer), 0.35 (2nd isomer)). Colorless solid, yield 7.23 mg (17%), mixture of two diastereomers (1:1). C_12_H_14_O_3_ (206.2). Exact mass (APCI): *m*/*z* = 207.1014, calcd. 207.1016 for C_12_H_15_O_3_ [MH]^+^. ^1^H-NMR (400 MHz, DMSO-*d*_6_): δ [ppm] = 1.52–1.93 (m, 2.5H, 4-*H_eq_* (1H), C*H_2_*CH_2_OH (1.5H)), 2.00 (m, 1.5H, C*H_2_*CH_2_OH (0.5H), 4-*H_ax_* (1H)), 3.66–3.81 (m, 2H, CH_2_C*H_2_*OH), 3.82–3.93 (m, 1H, 3-*H_ax_* (0.5H), 3-*H_eq_* (0.5H)), 4.61 (d, *J* = 4.4 Hz, 0.5H, CH_2_CH_2_O*H*), 4.73 (d, *J* = 5.7 Hz, 0.5H, CH_2_CH_2_O*H*), 5.33 (d, *J* = 10.8 Hz, 0.5H, 5-*H_eq_*), 5.41 (dd, *J* = 7.9/3.6 Hz, 0.5H, 5-*H_eq_*), 6.05 (s, 0.5H, 1-*H_eq_*), 6.09 (s, 0.5H, 1-*H_eq_*), 7.26–7.44 (m, 4H, H_arom_). Ratio of diastereomers 50:50. FTIR (neat): ν (cm^−1^) = 3372 (O-H), 2874 (C-H), 1462 (C=C_arom_), 1080 (C-O), 748 (C-H_arom 1,2-disubst_).

*2-[{(1RS,3RS,5RS)- and (1RS,3SR,5RS)-1-Methyl}-1,5-epoxy-1,3,4,5-tetrahydro-2-benzoxepin-3-yl]ethan-1-ol* (**14b**). A solution of diols **13b** (50.0 mg, 0.2 mmol) in a mixture of toluene/AcOH (4:1, 5mL) was stirred at r.t. for 72 h. Saturated aqueous Solution of NaHCO_3_ (5 mL) was added. The mixture was extracted with CH_2_Cl_2_ (3 × 10 mL). The combined organic layer was washed with brine (5 mL), dried (Na_2_SO_4_), filtered, and the solvent was removed in vacuo. The crude product was purified by fc (Ø = 2 cm, h = 10 cm, ethyl acetate/cyclohexane (1:1)), V = 10 mL, *R*_f_ = 0.45 (1st spot) and 0.25 (2nd spot)). Yellow oil, yield 2.6 mg (6%) mixture of two diastereomers (1:1). C_14_H_20_O_4_ (220.27). ^1^H-NMR (400 MHz, CDCl_3_): δ [ppm] = 1.26 (2s, 3H, 6 × 0.5H, C*H_3_*), 1.62–1.89 (m, 1H, 4-*H_eq_* (0.5H) *, C*H_2_*CH_2_OH (0.5H)), 1.89–2.15 (m, 1.5H, C*H_2_*CH_2_OH), 2.17–2.33 (m, 1H, 2 × 0.5H, 4-*H_ax_*), 3.88 (dd, *J* = 12.0/4.7 Hz, 1H**, 2 × 0.5H, CH_2_C*H_2_*OH), 3.94–3.98 (m, 1H **, 2 × 0.5H, CH_2_C*H_2_*OH), 4.01–4.09 (m, 0.5H, 3-*H_ax_*), 4.08–4.14 (m, 0.5H, 3-*H_ax_*), 5.31–5.36 (m, 0.5H, 5-*H_eq_*), 5.67 (dd, *J* = 6.1/2.7 Hz, 0.5H, 5-*H_eq_*), 7.19 (m, 1H, 2 × 0.5H, H_arom_), 7.29–7.47 (m, 3H, 6 × 0.5H, H_arom_). Ratio of diastreoisomers 50:50. * Signals for one isomer is overlapping with other signals including the signal for the residual water in CDCl_3_. ** Signals are slightly higher than expected due to impurities. Due to the low amount of **18b** the ^13^C-NMR, IR and MS spectra could not be recorded.

*(1RS,3SR,5RS)-3-[2-(tert-Butyldimethylsilyloxy)ethyl]-1,5-epoxy-1,3,4,5-tetrahydro-2-benzoxepine* (**22a**). In toluene (5 mL), silyl ethers **19a** (26.6 mg, 0.08 mmol) were treated with 0.1 M HCl in AcOH (15 μL) and was heated to 65 °C for 60 h. The reaction mixture was diluted with EtOAc (10 mL) and was washed with saturated aqueous solution of NaHCO_3_ (3 × 5 mL). The pooled organic phase was dried (Na_2_SO_4_), filtered, and the solvent was removed in vacuo. The crude product was purified by fc (Ø = 2 cm, h = 10 cm, cyclohexane: ethyl acetate (9:1), V = 10 mL, *R*_f_ = 0.48). Colorless oil, yield 4.5 mg (19%). C_18_H_28_O_3_Si (320.5). ^1^H-NMR (400 MHz, DMSO-*d*_6_): δ [ppm] = −0.12 (s, 3H, (C*H_3_*)_2_^t^BuSi), −0.14 (s, 3H, (C*H_3_*)_2_^t^BuSi), 0.68 (s, 9H, Me_2_ (C(C*H_3_*)_3_Si), 1.44 (ddd, *J* = 13.8/7.7/1.9 Hz, 1H, 4-H_eq_), 1.50–1.62 (m, 2H, C*H_2_*CH_2_OTBDMS), 2.01 (ddd, *J* = 13.2/11.2/3.7 Hz, 1H, 4-H_ax_), 3.20–3.28 (m, 1H, 3-H_ax_), 3.44–3.53 (m, 2H, C*H_2_*OTBDMS), 5.23 (dd, *J* = 3.7/1.8 Hz, 1H, 5-H_eq_), 6.12 (s, 1H, 1-H_eq_), 7.25–7.37 (m, 4H, H_arom_).

*(1RS,3SR,5RS)-3-(2-Azidoethyl)-1,5-epoxy-1,3,4,5-tetrahydro-2-benzoxepine* (**23a**). A solution of *p*-toluenesulfonic acid in toluene (5 mL, 0.5 mg/mL) was added to azide **21a** (69.8 mg, 0.27 mmol). The reaction mixture was heated to 85 °C for 72 h. Saturated aqueous solution of NaHCO_3_ (10 mL) was added and the organic layer was separated. The aqueous layer was extracted with CH_2_Cl_2_ (2 × 10 mL). The combined organic layer was dried (Na_2_SO_4_), filtered, and the solvent was removed in vacuo. The crude product was purified by fc (Ø = 2 cm, h = 12 cm, cyclohexane: ethyl acetate (8:2), V = 10 mL, *R*_f_ = 0.45). Yellow oil, yield 7.7 mg (13%). C_12_H_13_N_3_O_2_ (231.3). After fc purification, only one diastereomer was isolated. MS (ESI): *m*/*z* =424 [2(M – OCH3) + Na + H]^+^, 232 [MH]^+^, 204 [M –N_2_ + H]^+^. ^1^H-NMR (400 MHz, CD_2_Cl_2_): δ [ppm] = 1.54–1.66 (m, 2H, 4-*H_eq_* (1H), C*H_2_*CH_2_N_3_ (1H)), 1.76 (ddt, *J* = 14.5/8.7/5.8 Hz, 1H, C*H_2_*CH_2_N_3_), 2.17 (ddd, *J* = 13.0/11.1/3.7 Hz, 1H, 4-*H_ax_*), 3.22–3.35 (m, 3H, CH*_2_*C*H*_2_N_3_ (2H), 3-*H*_ax_ (1H)), 5.22 (dd, *J* = 3.8/1.8 Hz, 1H, 5-*H_eq_*), 6.11 (s, 1H, 1-*H_eq_*), 7.28–7.40 (m, 4H, H_arom_). ^13^C-NMR (151 MHz, CDCl_3_): δ [ppm] = 35.1 (1C, *C*H_2_CH_2_N_3_), 37.6 (1C, C-4), 47.2 (1C, CH_2_*C*H_2_N_3_), 65.3 (1C, C-3), 77.9 (1C, C-5), 100.8 (1C, C-1), 119.6 (1C, C_arom_), 121.3 (1C, C_arom_), 128.0 (1C, C_arom_), 128.6 (1C, C_arom_), 138.7 (1C, Cq_arom_), 143.0 (1C, Cq_arom_). FTIR (neat): ν (cm^−1^) = 2874 (C-H), 2095 (N=N=N), 1015 (C-O), 756 (C-H_arom 1,2-disubst_).

## 4. Conclusions

Herein the synthesis of 1,3-dioxanes is reported, which are bearing a phenyl moiety in the axial orientation at the acetalic center without any freedom to rotate around the phenyl-C-bond. Two subsequent intramolecular transacetalization reactions represent the key steps in the synthesis of the tricyclic compounds **14**, **22**, and **23**. The central hydroxyacetals **12** were prepared in a three-step sequence starting from pentane-1,3,5-triol (**10**). Whereas the first intramolecular transacetalization of **12** under mild reaction conditions resulted in high yields of 2-benzofurans **18**, the second intramolecular transacetalization of **13** gave only low yields of tricyclic alcohols **14** even after several optimization experiments. Protection or substitution of the competing primary alcohol of **13a** did not result in higher yields of tricyclic silyl ether **22a** or azide **23a**. Since only one isomer of **22a** and **23a** was isolated and the major isomer remained partly unchanged after cyclization of **19a** and **21a**, it was postulated that the minor isomers of **19a** and **21a** reacted faster than the major isomers. Due to fast decomposition, the transformations of ketalic compounds (**b**-series) required milder reaction conditions than the acetalic compounds (**a**-series).

## Supplementary Materials

Supplementary materials can be accessed at: http://www.mdpi.com/1420-3049/21/11/1503/s1.
