# Supplementary material for: Double Intramolecular Transacetalization of Polyhydroxy Acetals: Synthesis of Conformationally-Restricted 1,3-Dioxanes with Axially-Oriented Phenyl Moiety
Source: Molecules. 2016 Nov 9;21(11):1503. doi: 10.3390/molecules21111503 (PMC6274033; doi:10.3390/molecules21111503)
Supplement: Supplementary file 1 [file molecules-21-01503-s001.pdf]

Supplementary Materials: Double Intramolecular Transacetalization of Polyhydroxy Acetals: Synthesis of Conformationally Restricted 1,3-Dioxanes with Axially Oriented Phenyl Moiety

Samuel Asare-Nkansah and Bernhard Wünsch

The Supplementary Materials contains representative ^{1}H- and ^{13}C-NMR spectra.

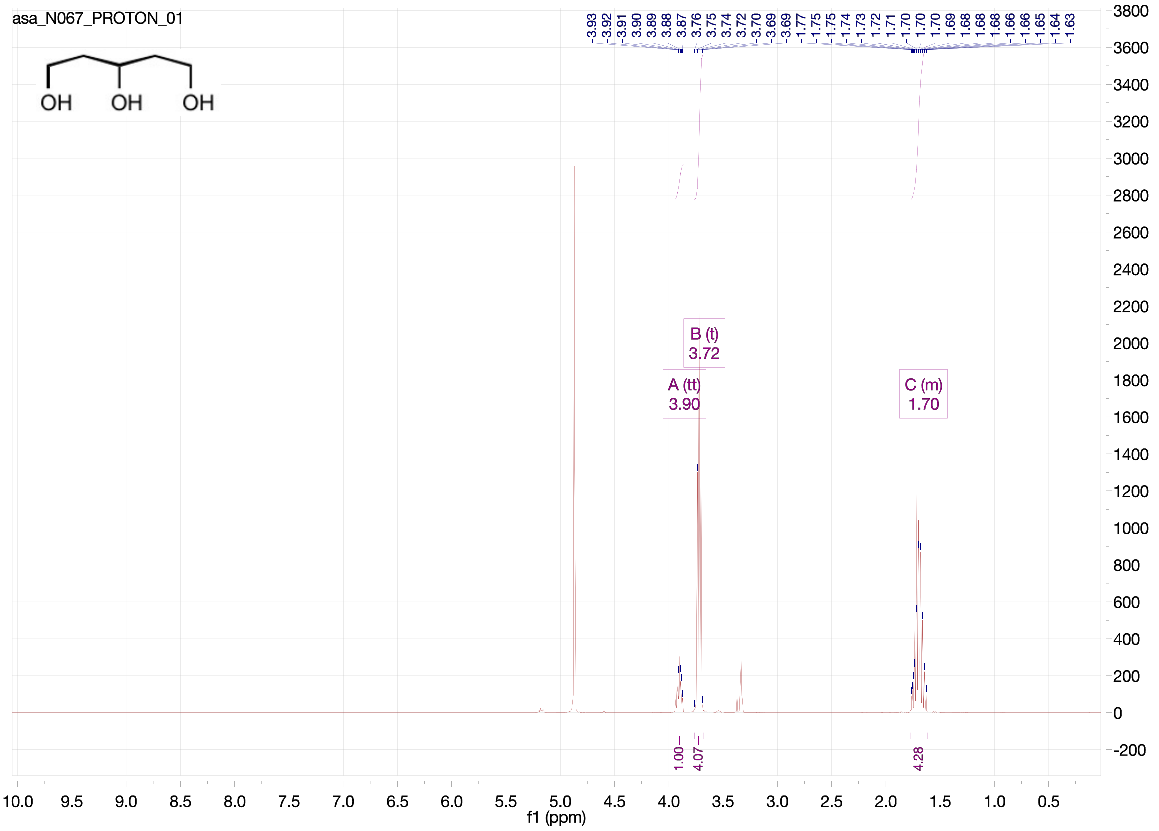

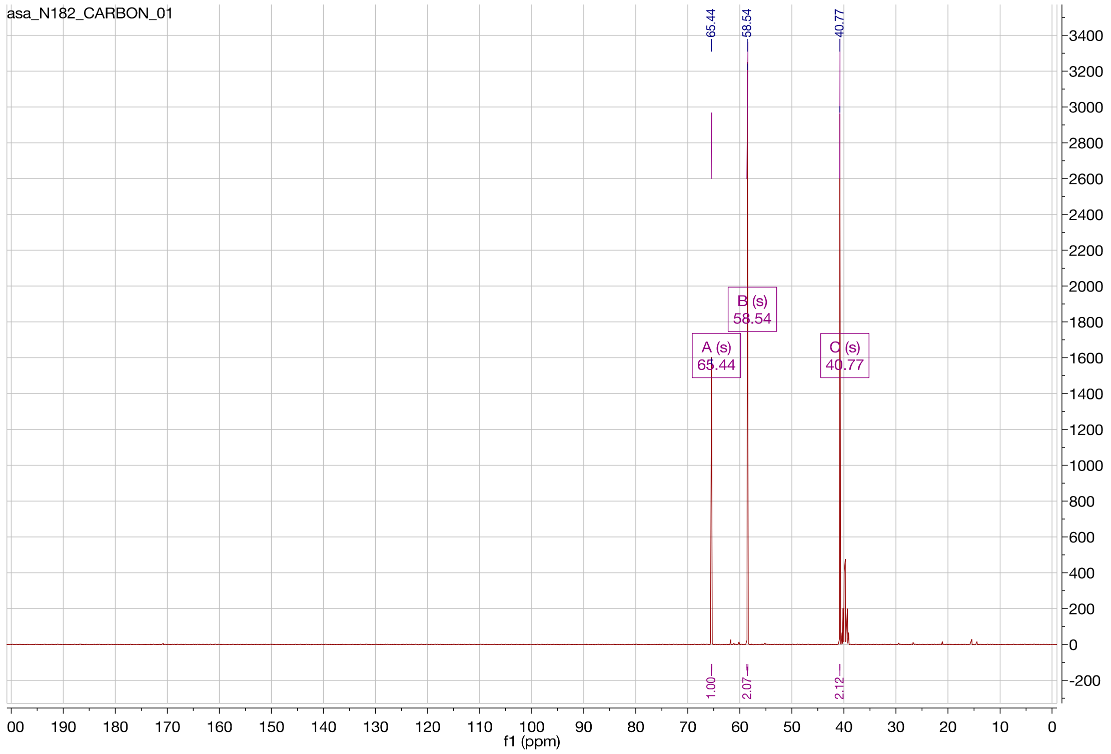

Figure S1. ^{1}H- (top) and ^{13}C-NMR spectrum (bottom) of **10**.

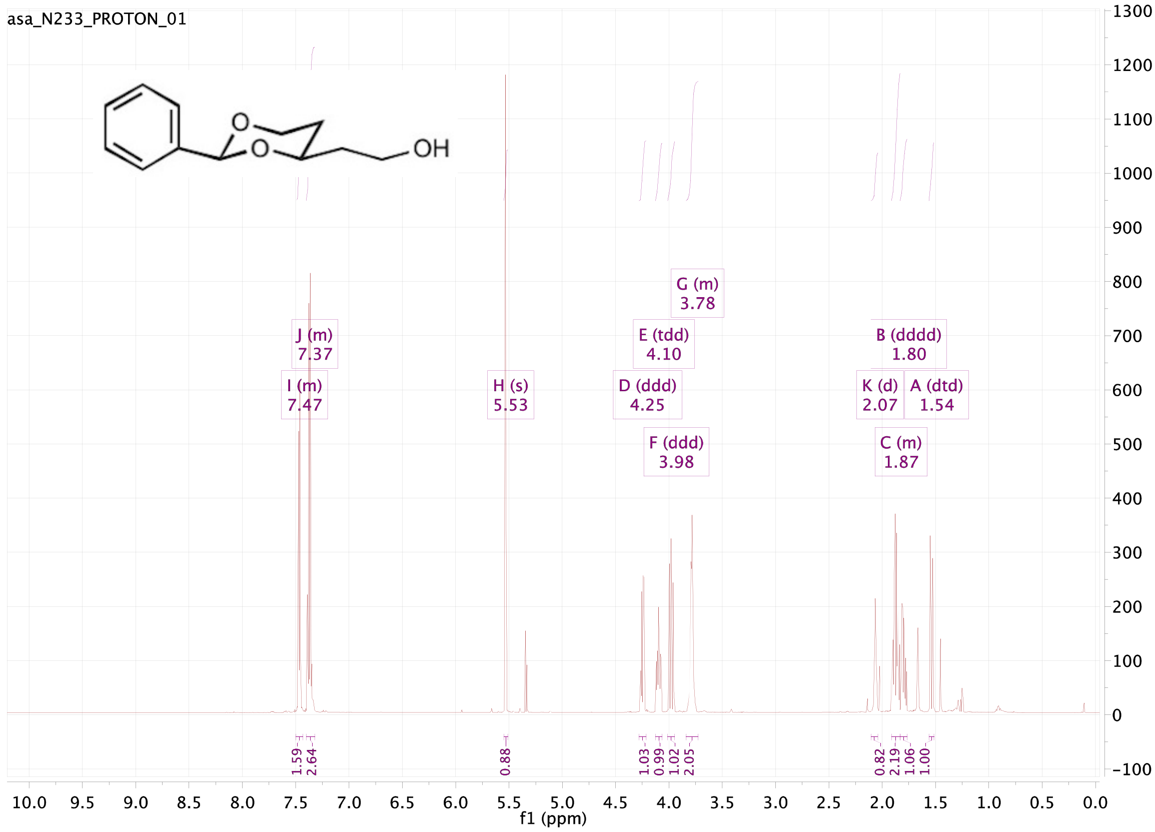

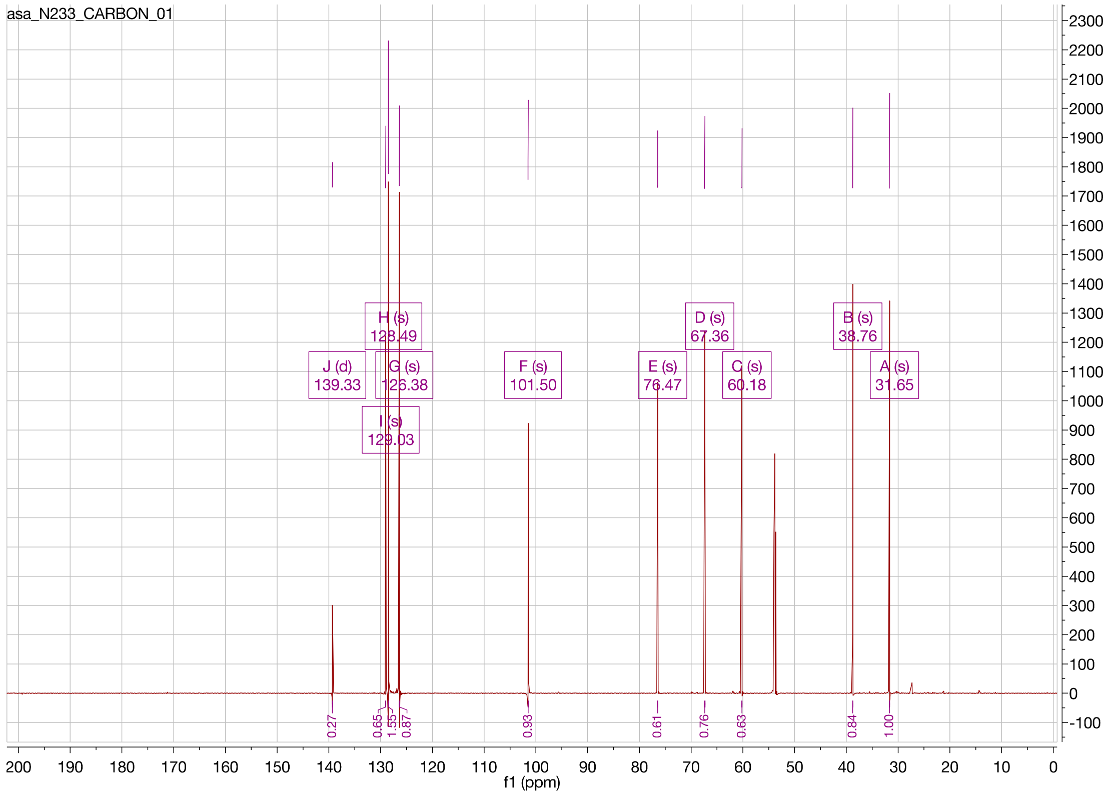

Figure S2. ^{1}H- (top) and ^{13}C-NMR spectrum (bottom) of **16**.

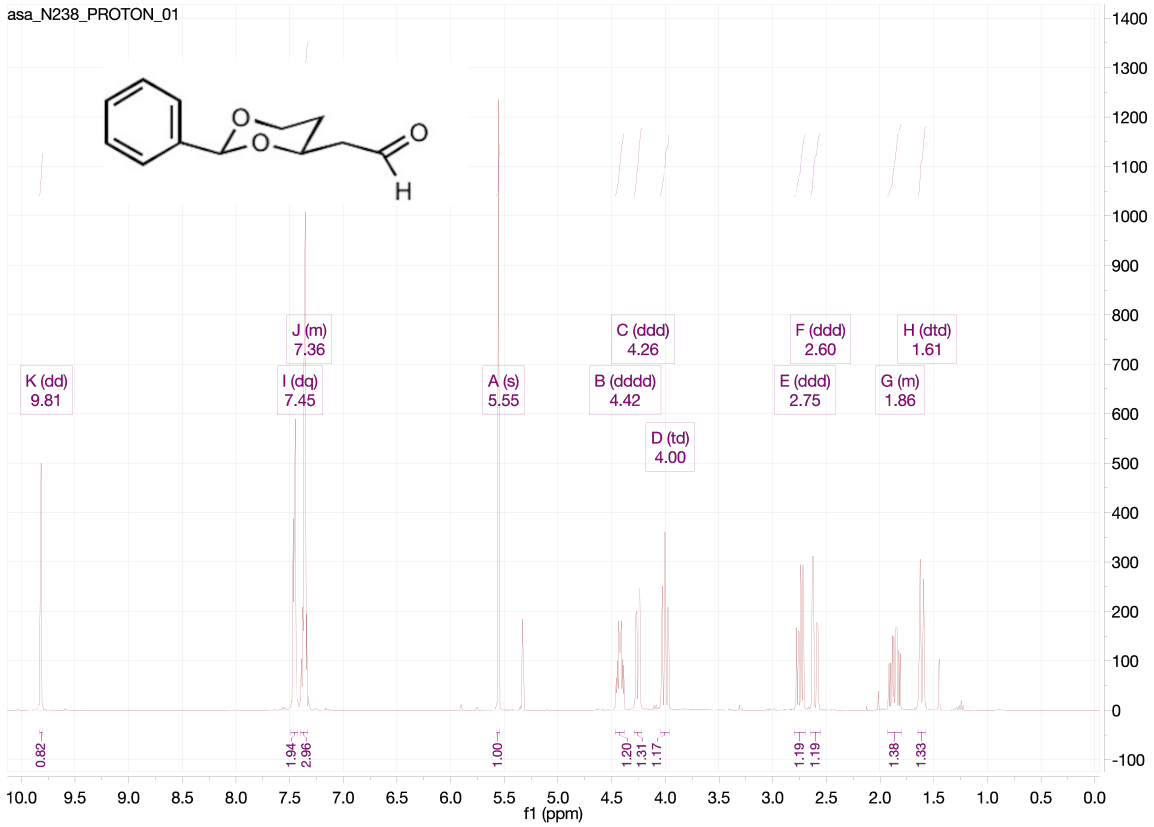

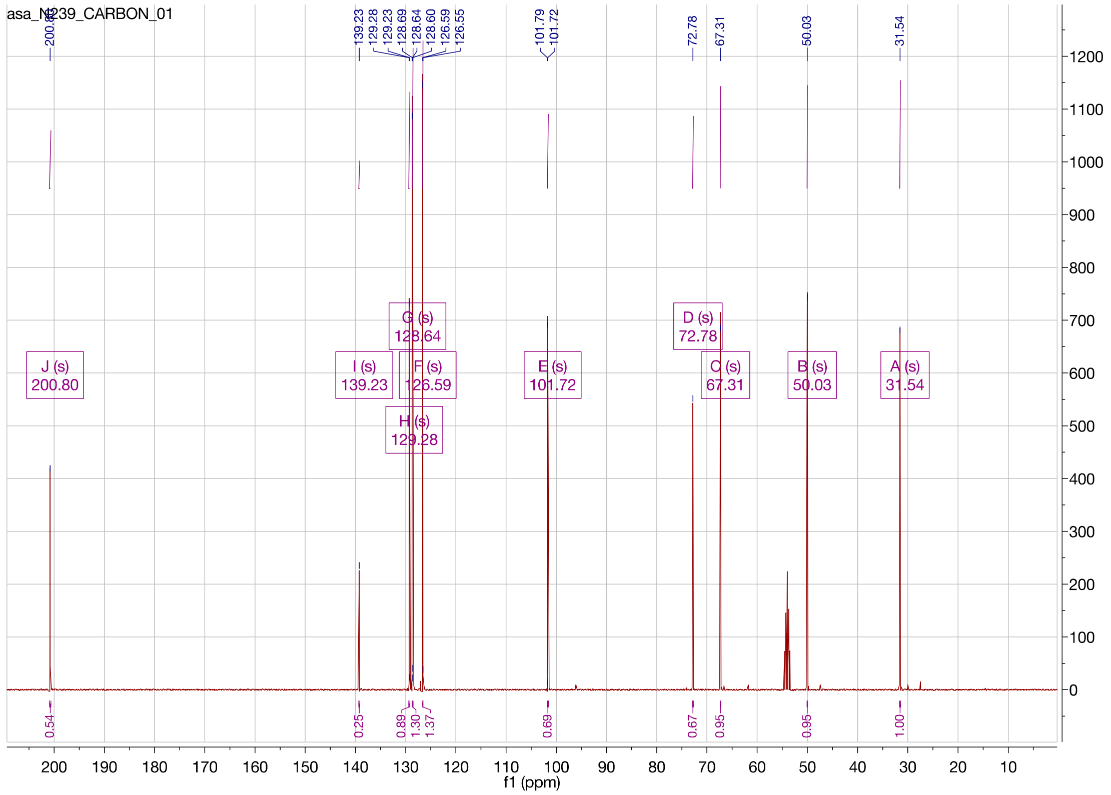

Figure S3. ^{1}H- (top) and ^{13}C-NMR spectrum (bottom) of **11**.

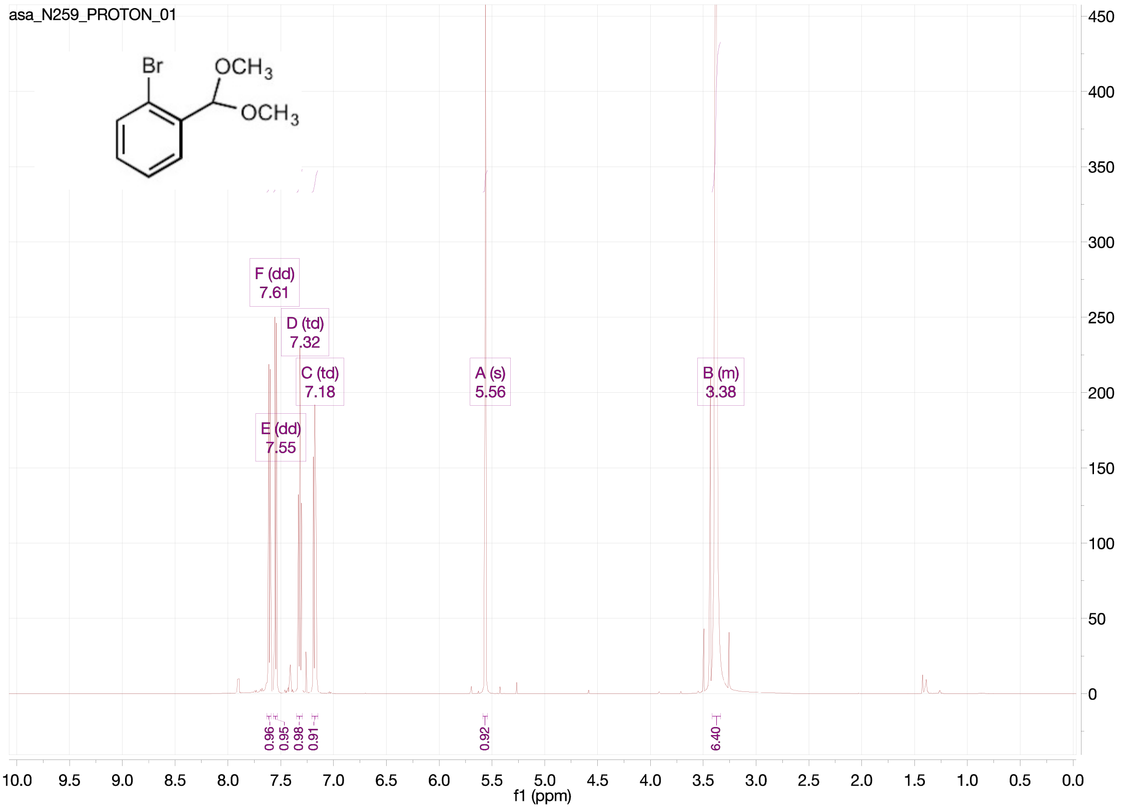

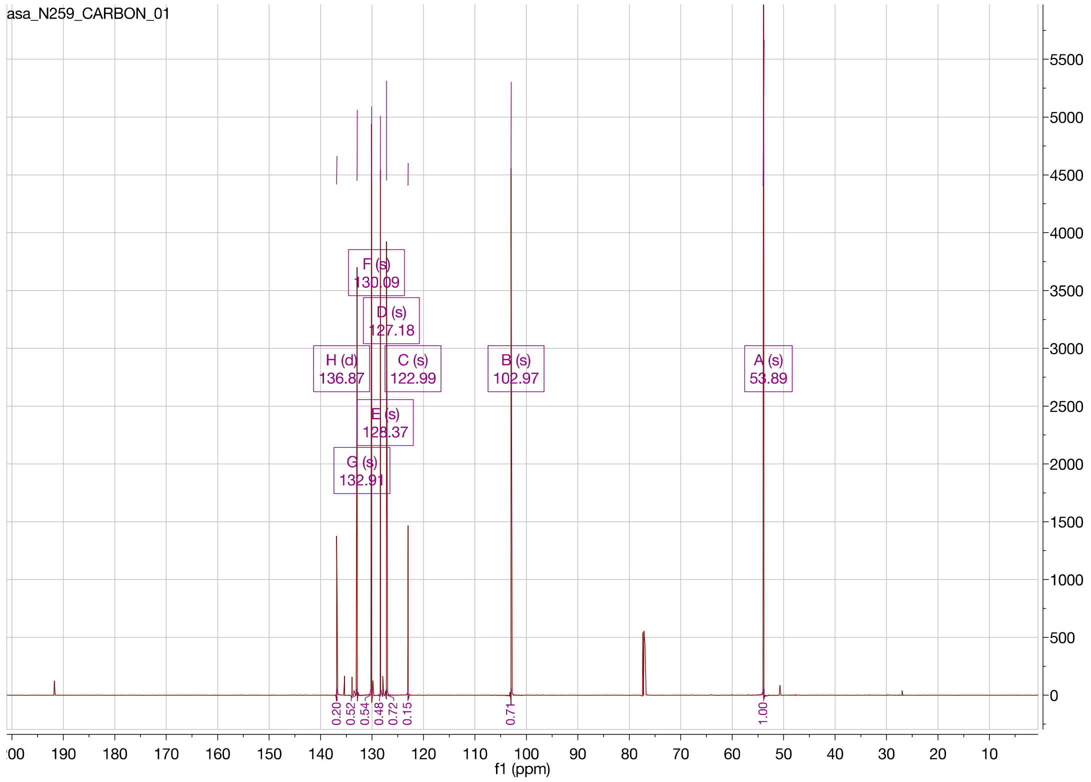

Figure S4. ^{1}H- (top) and ^{13}C-NMR spectrum (bottom) of **17a**.

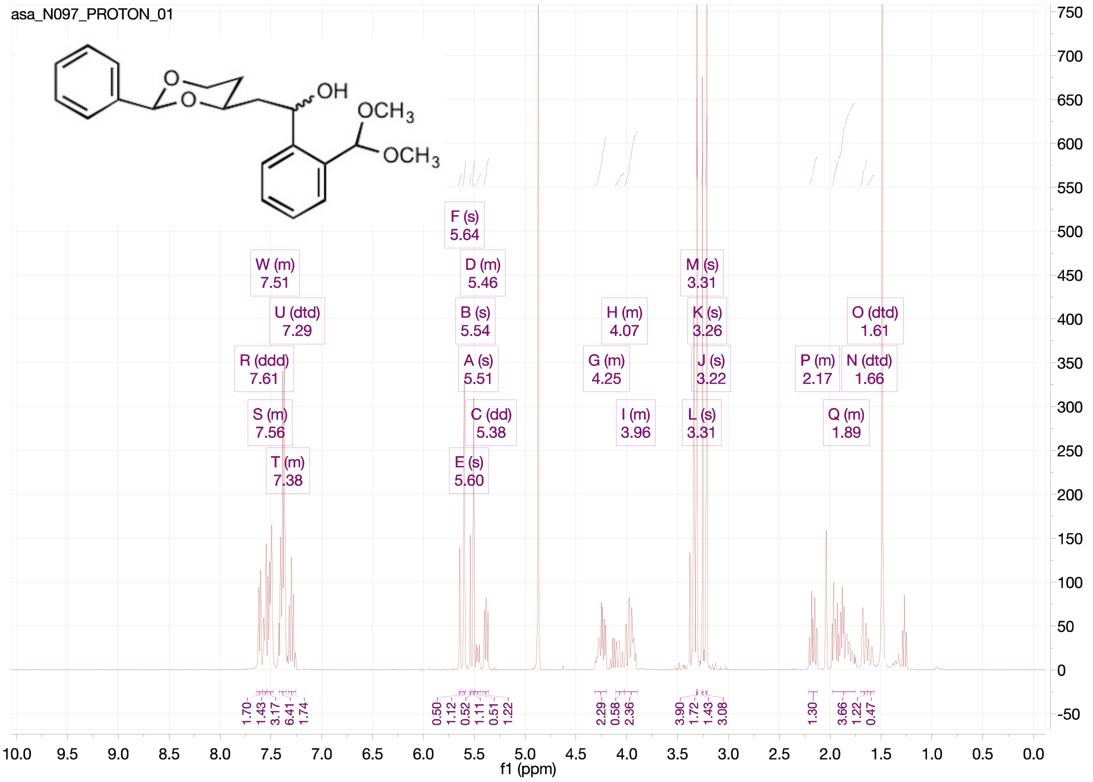

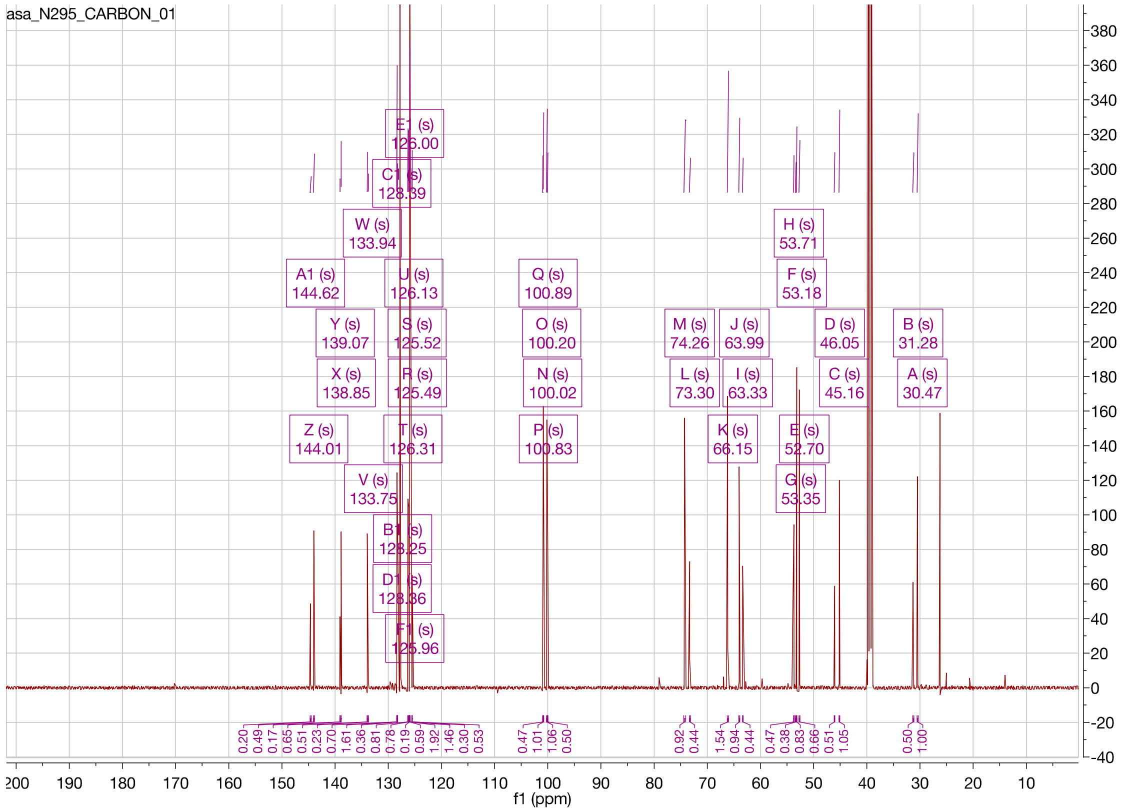

Figure S5. ¹H- (top) and ¹³C-NMR spectrum (bottom) of **12a**.

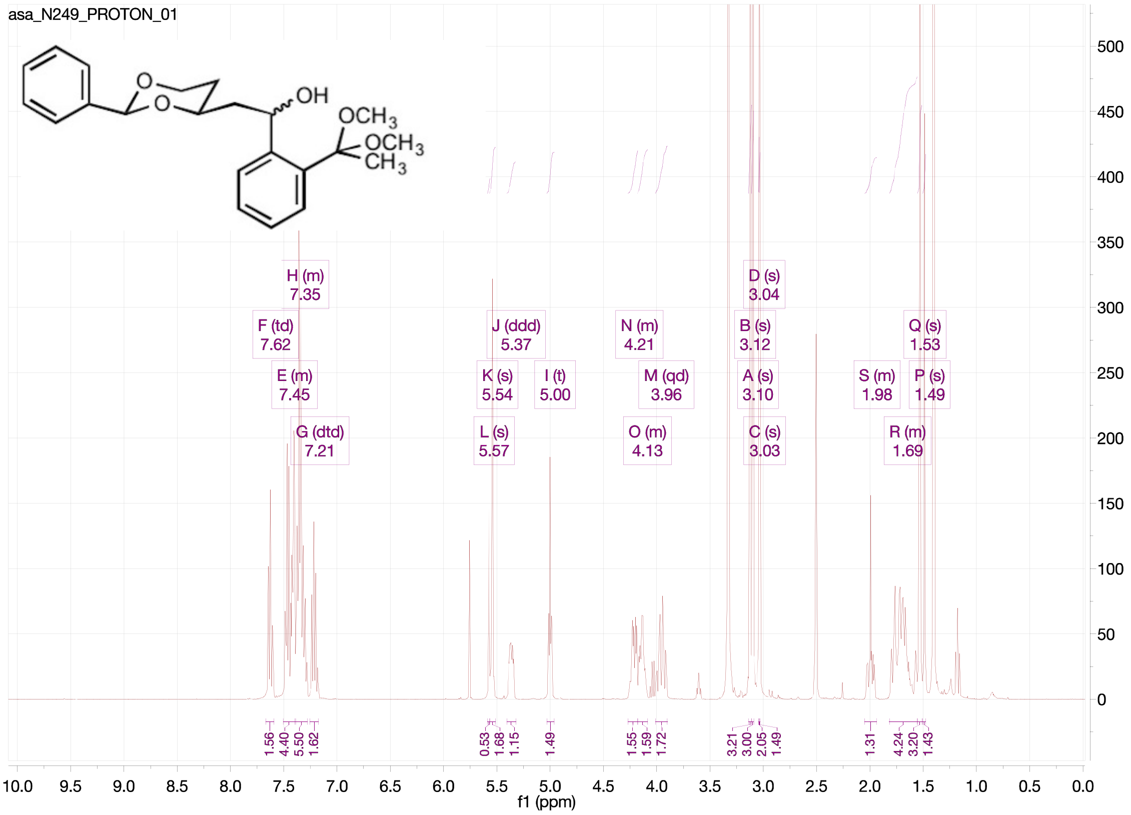

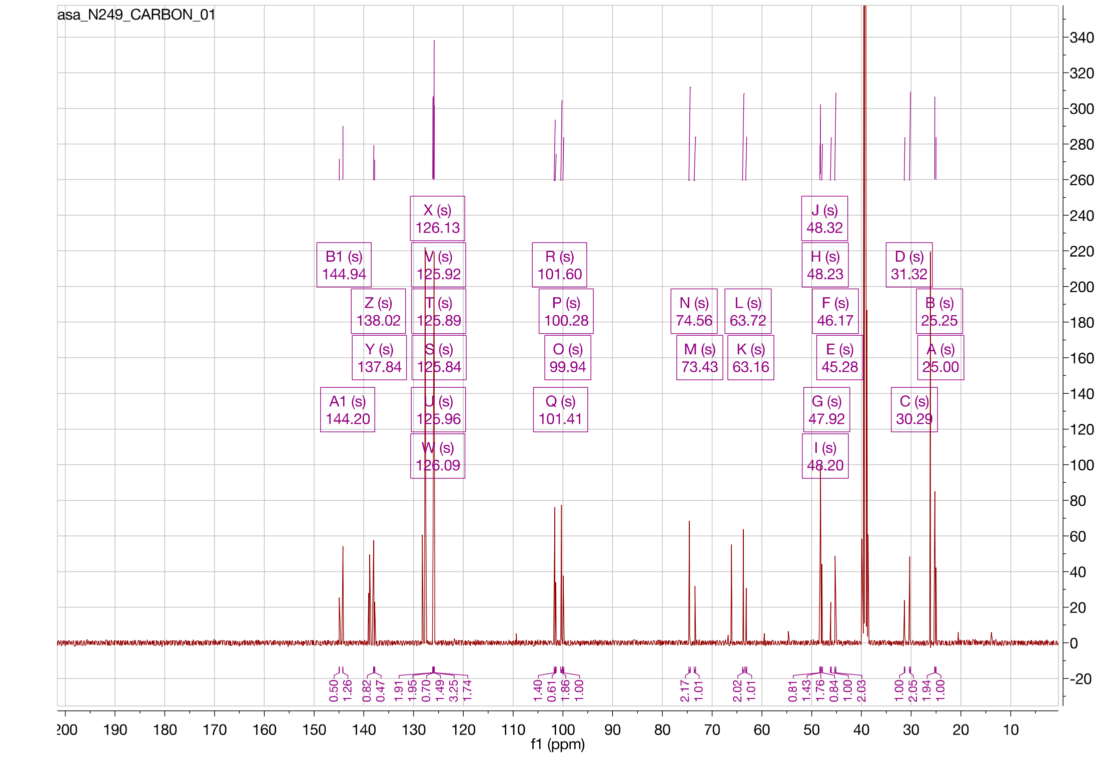

Figure S6. ^{1}H- (top) and ^{13}C-NMR spectrum (bottom) of **12b**.

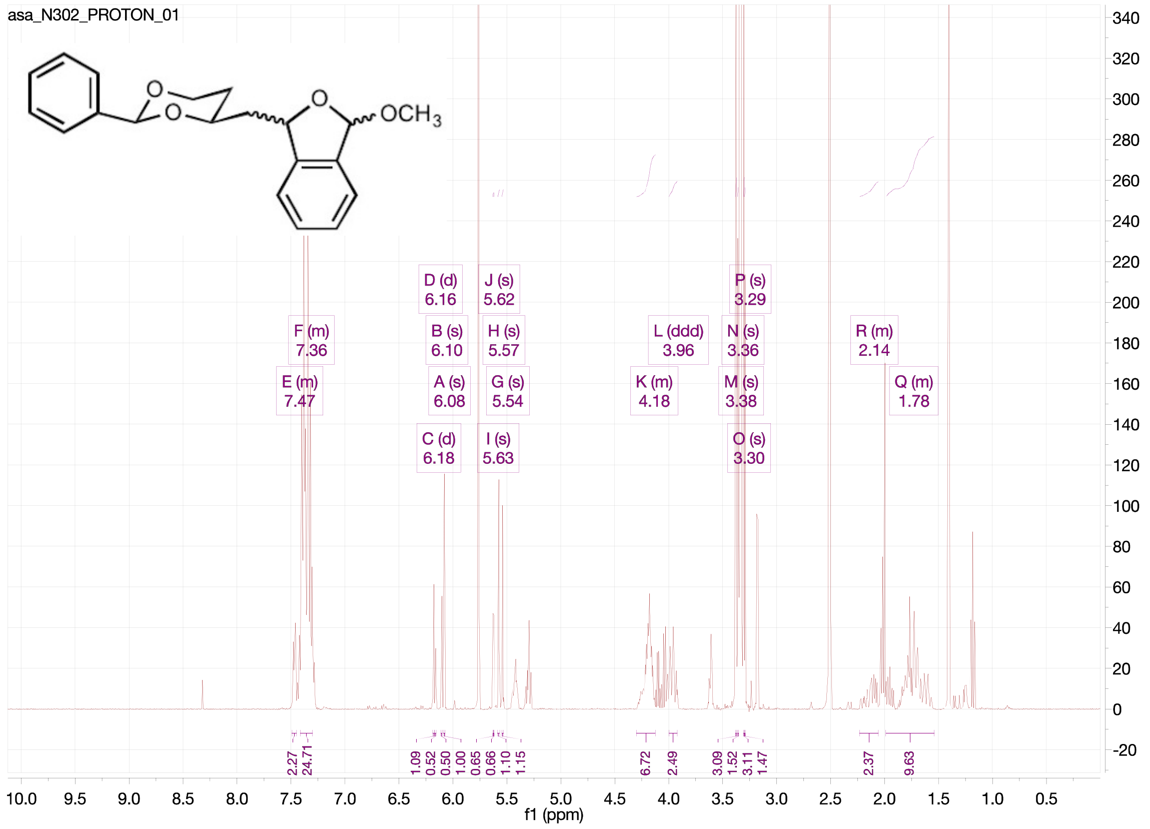

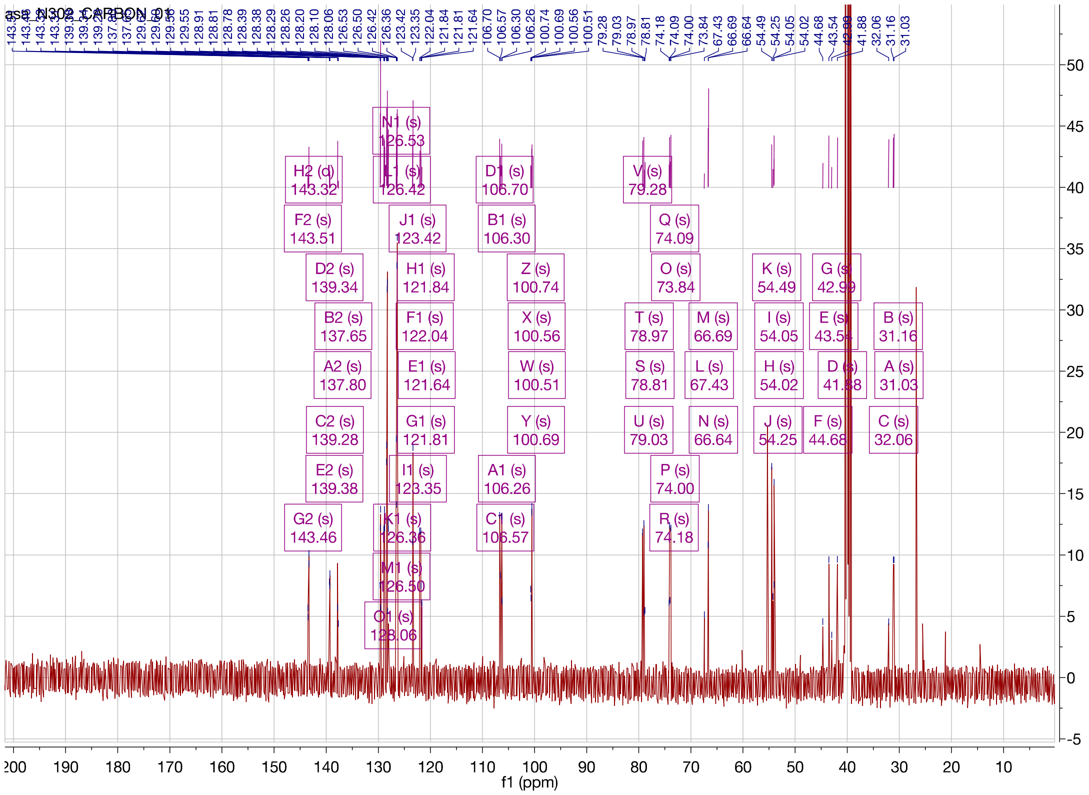

Figure S7. ^{1}H- (top) and ^{13}C-NMR spectrum (bottom) of **18a**.

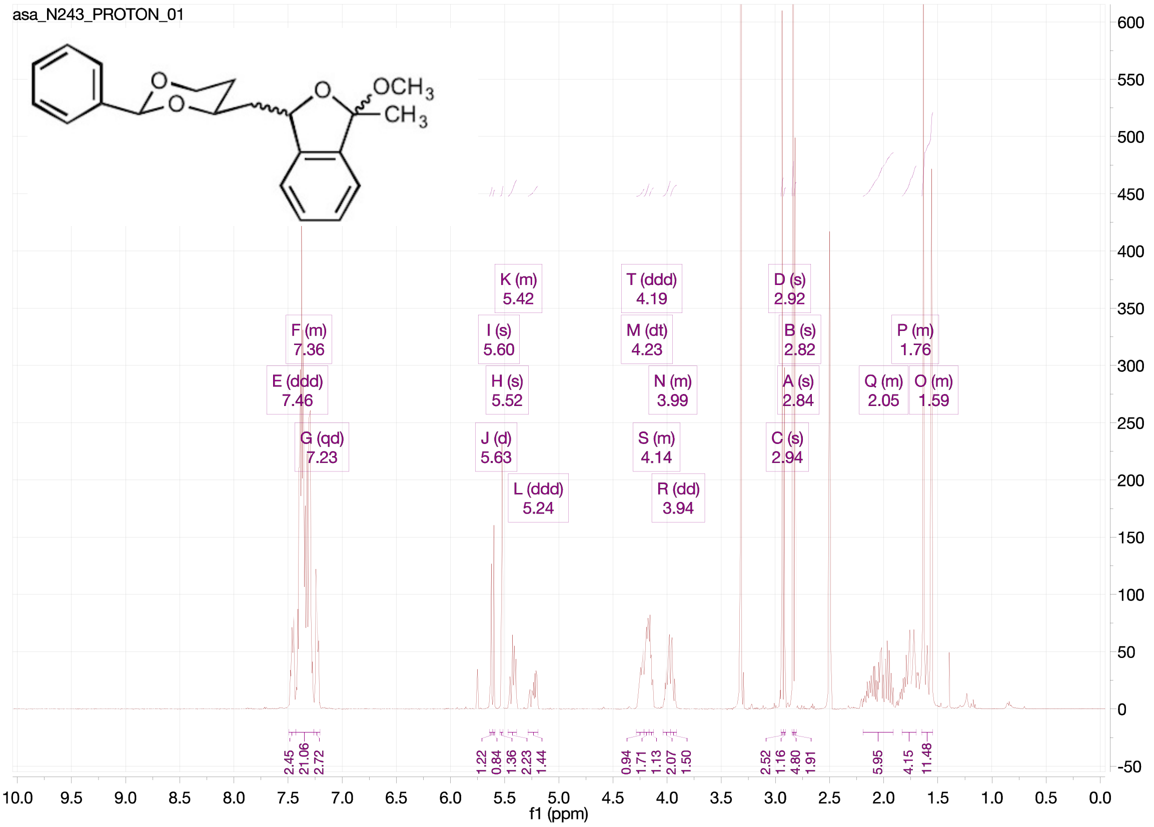

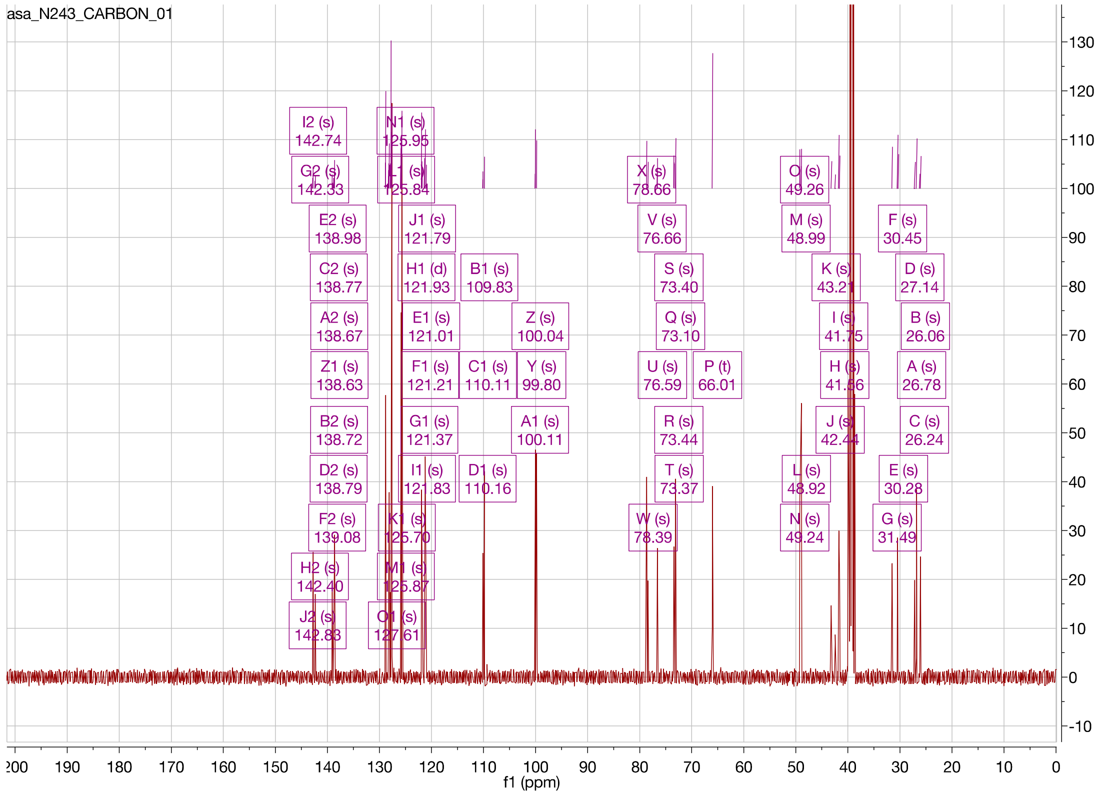

Figure S8. ^{1}H- (top) and ^{13}C-NMR spectrum (bottom) of **18b**.

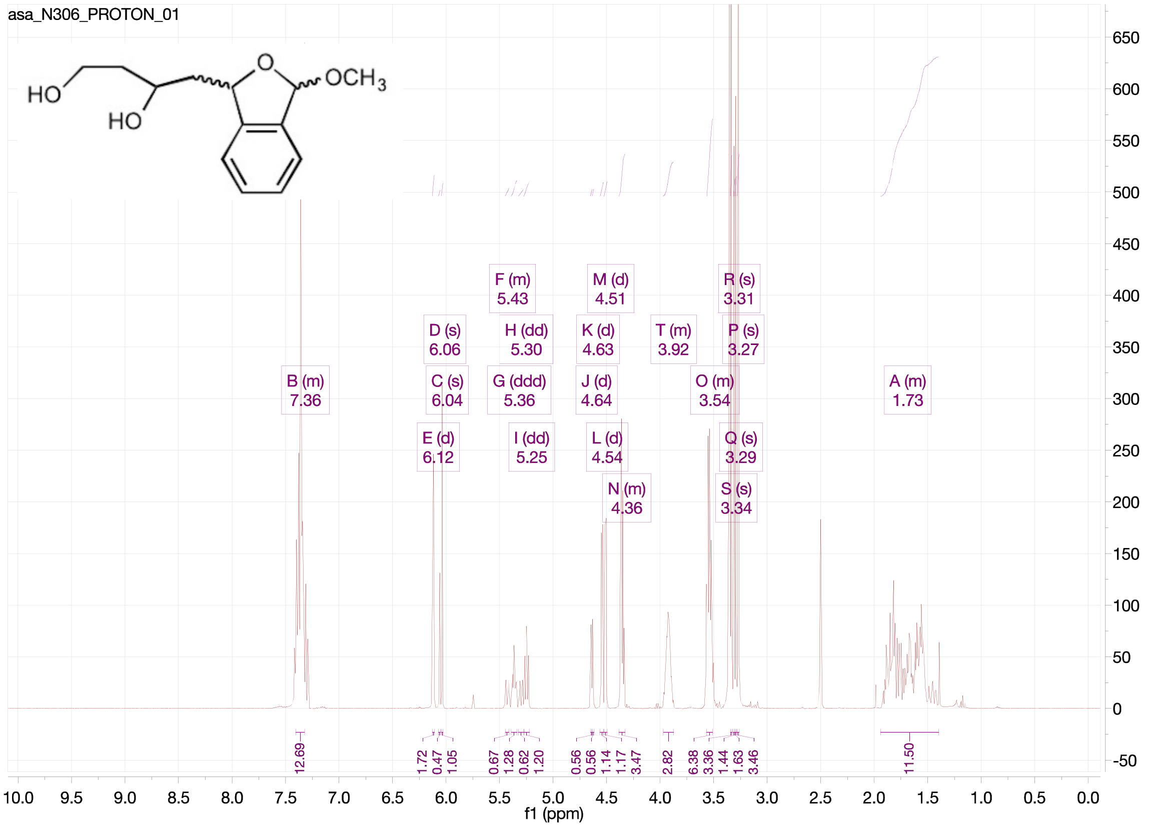

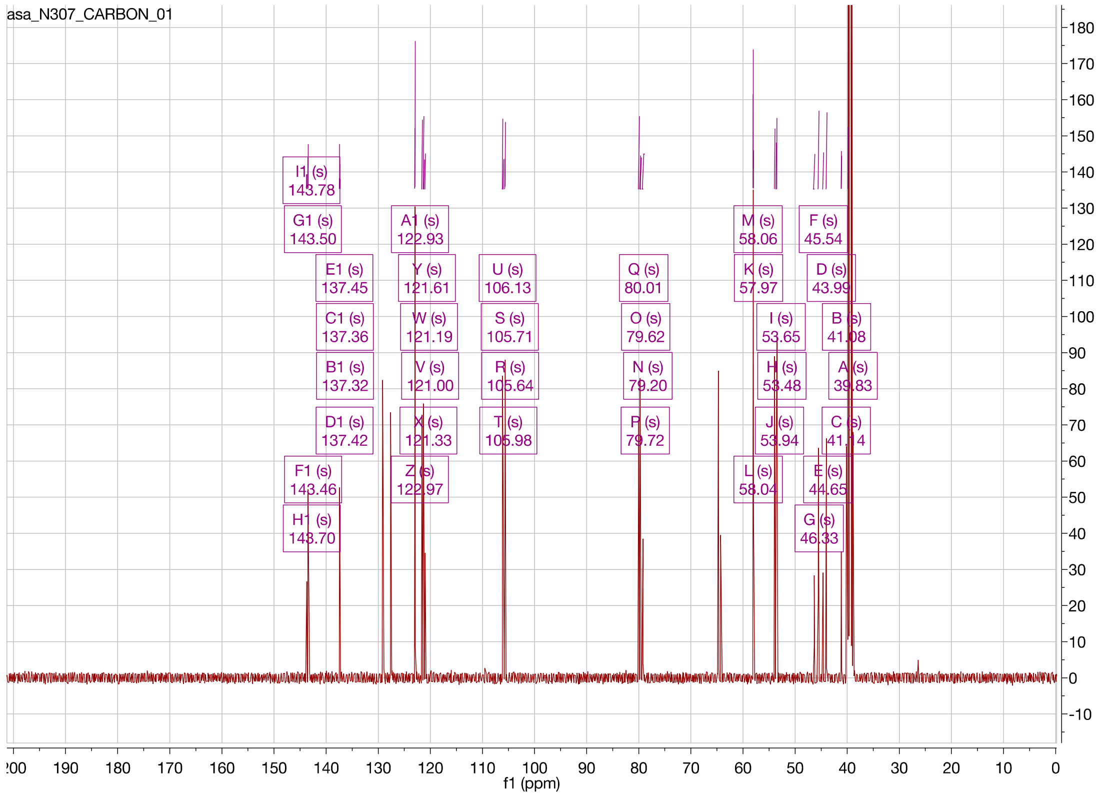

Figure S9. ^{1}H- (top) and ^{13}C-NMR spectrum (bottom) of **13a**.

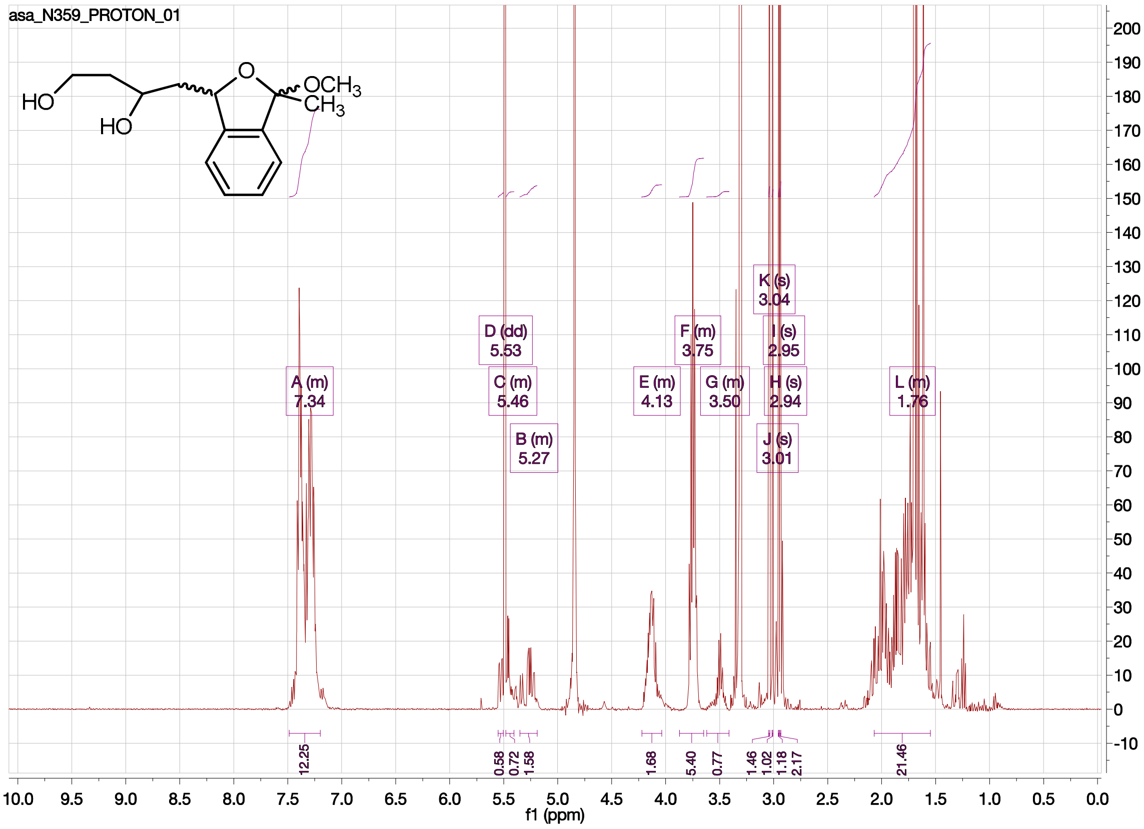

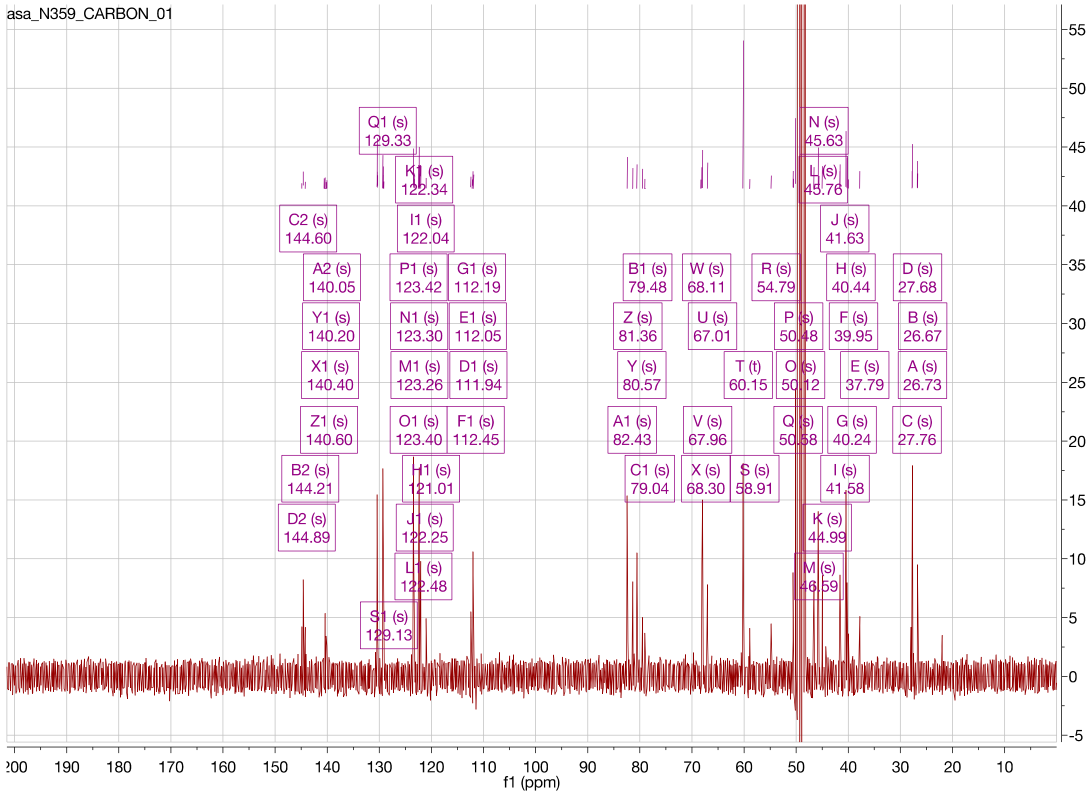

Figure S10. ^{1}H- (top) and ^{13}C-NMR spectrum (bottom) of **13b**.

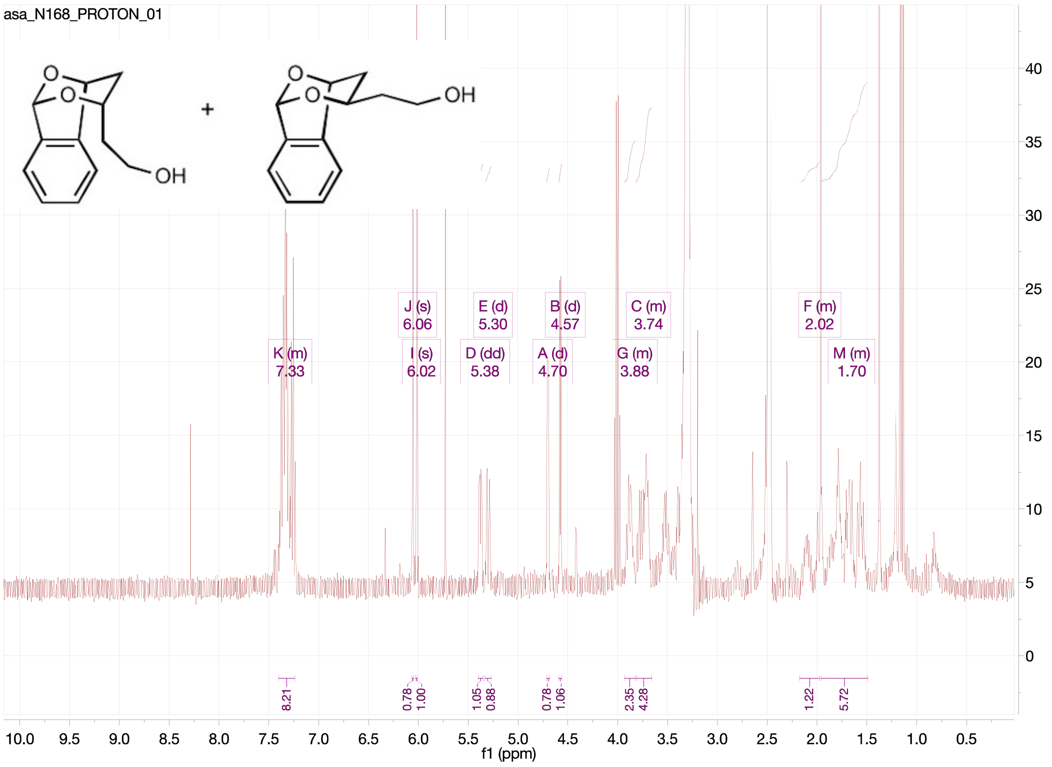

Figure S11. ^{1}H-NMR spectrum of **14a**.

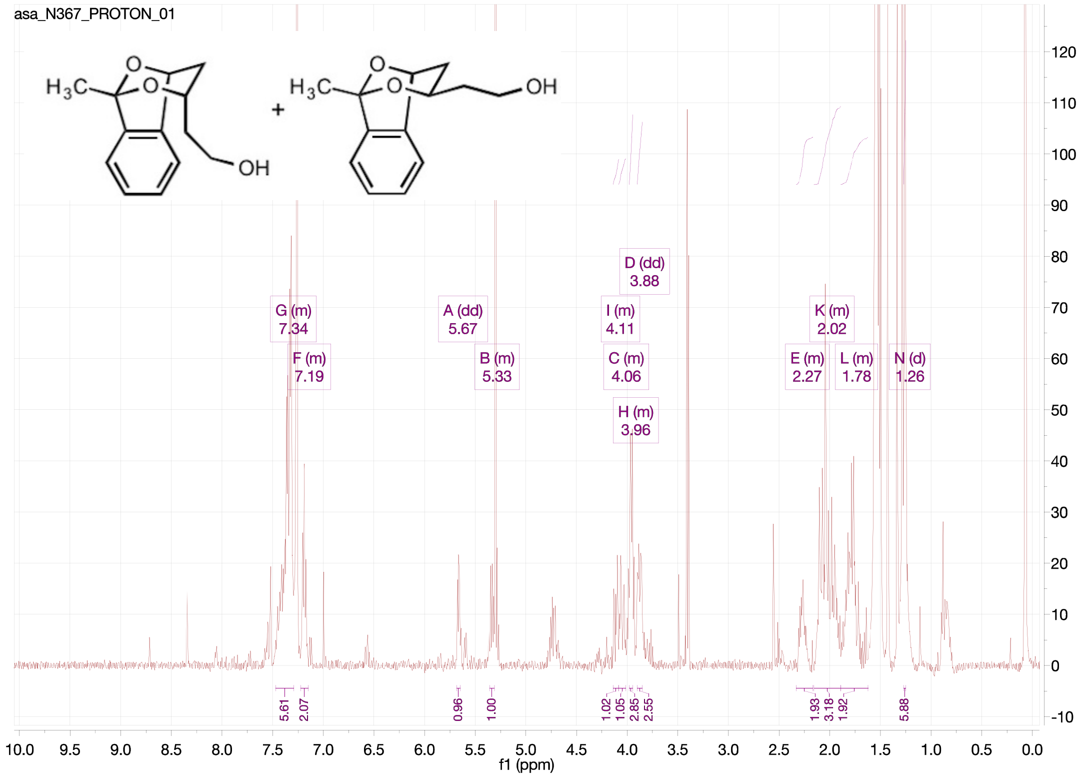

Figure S12. ^{1}H-NMR spectrim of **14b**.

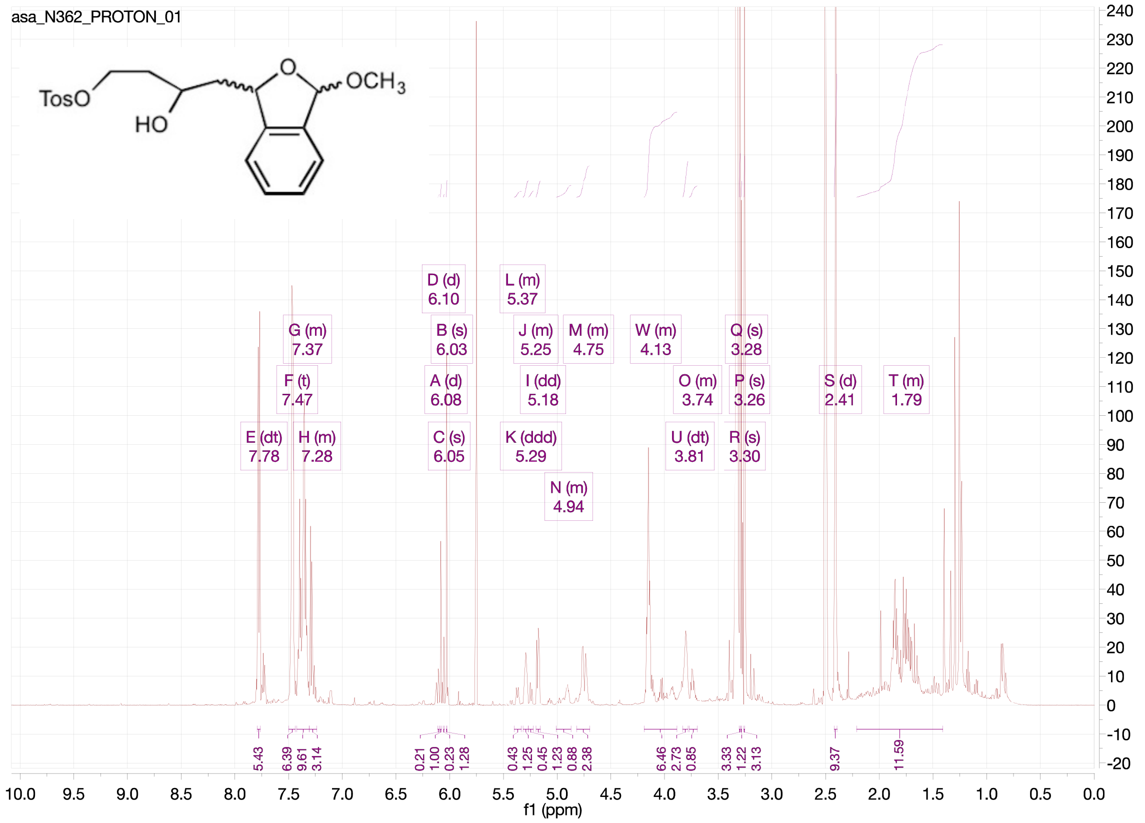

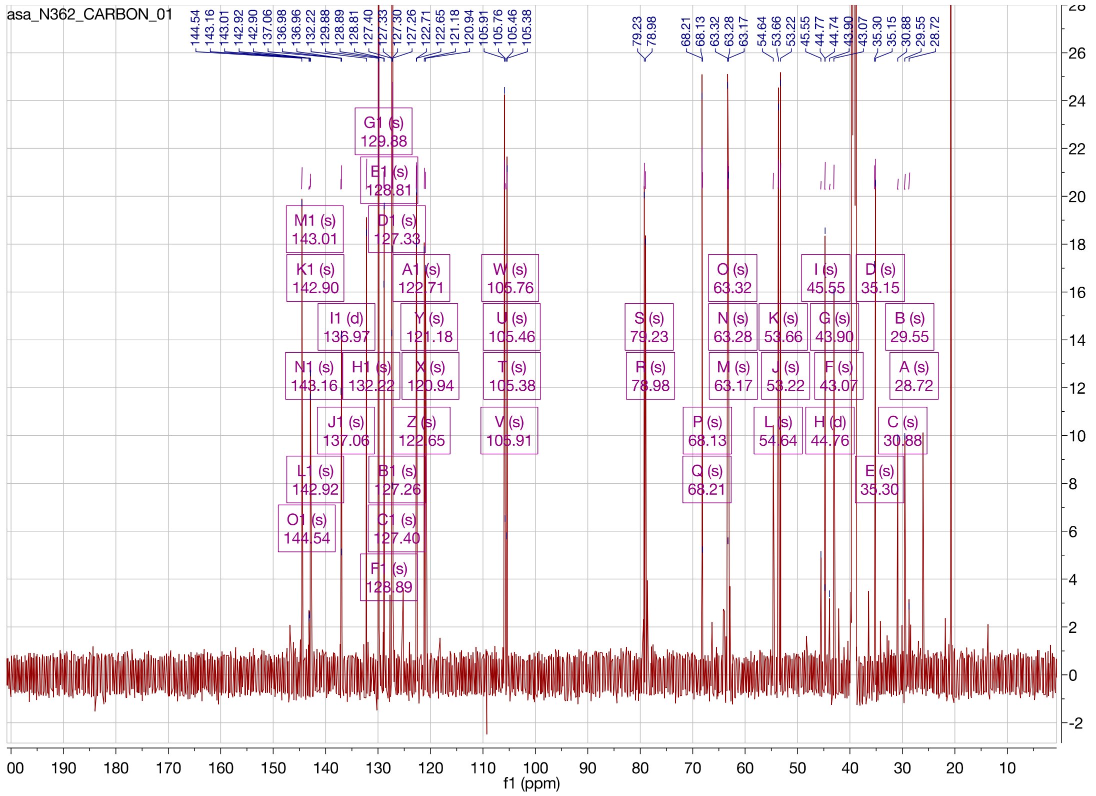

Figure S13. ^{1}H- (top) and ^{13}C-NMR spectrum (bottom) of **20a**.

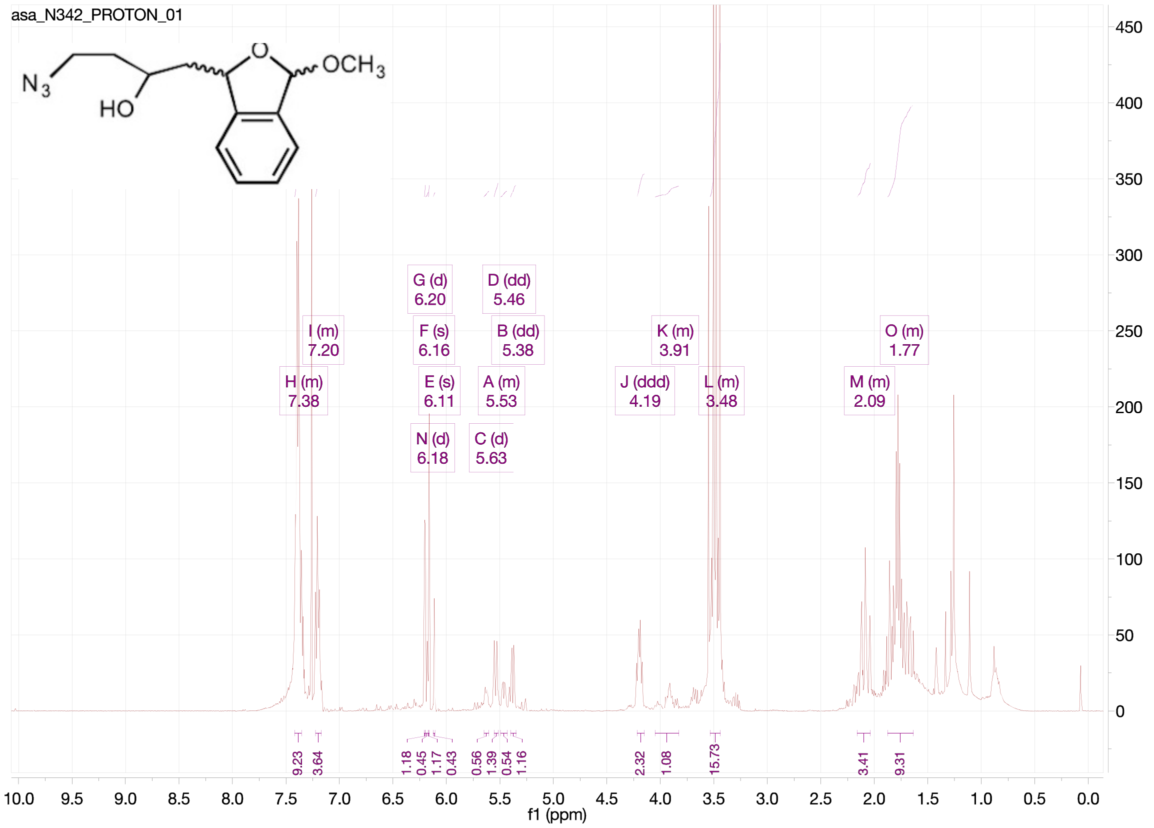

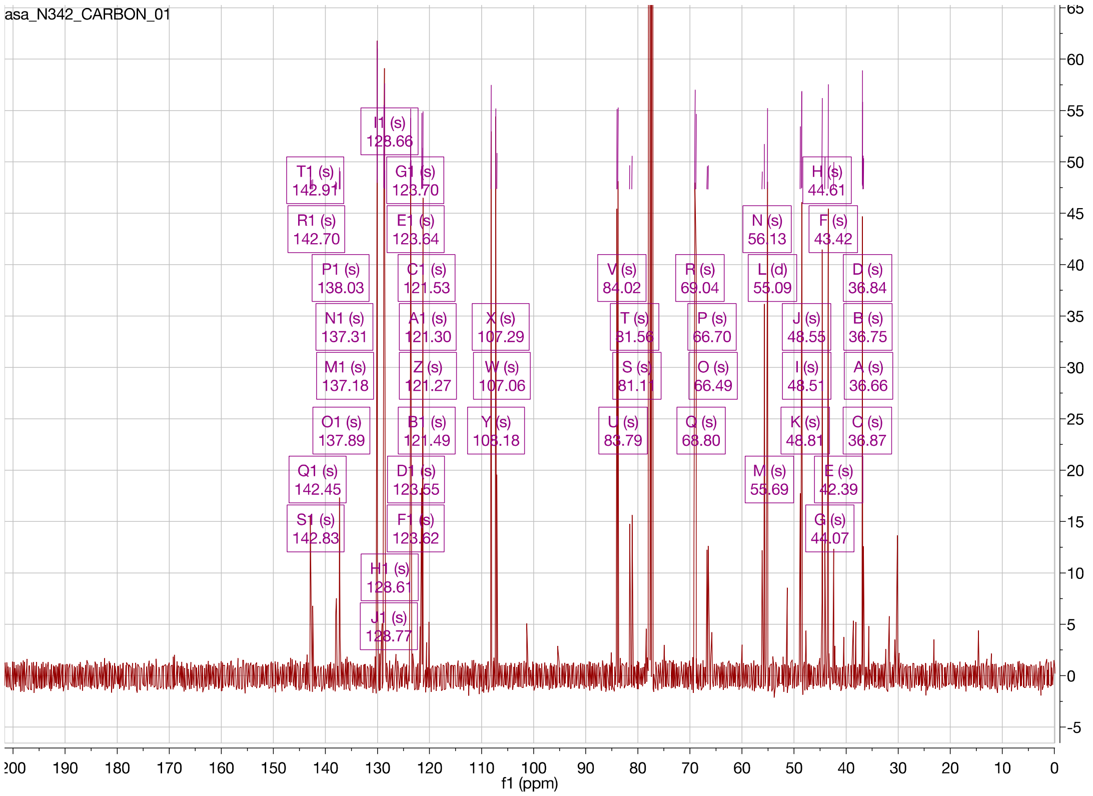

Figure S14. ¹H- (top) and ¹³C-NMR spectrum (bottom) of **21a**.

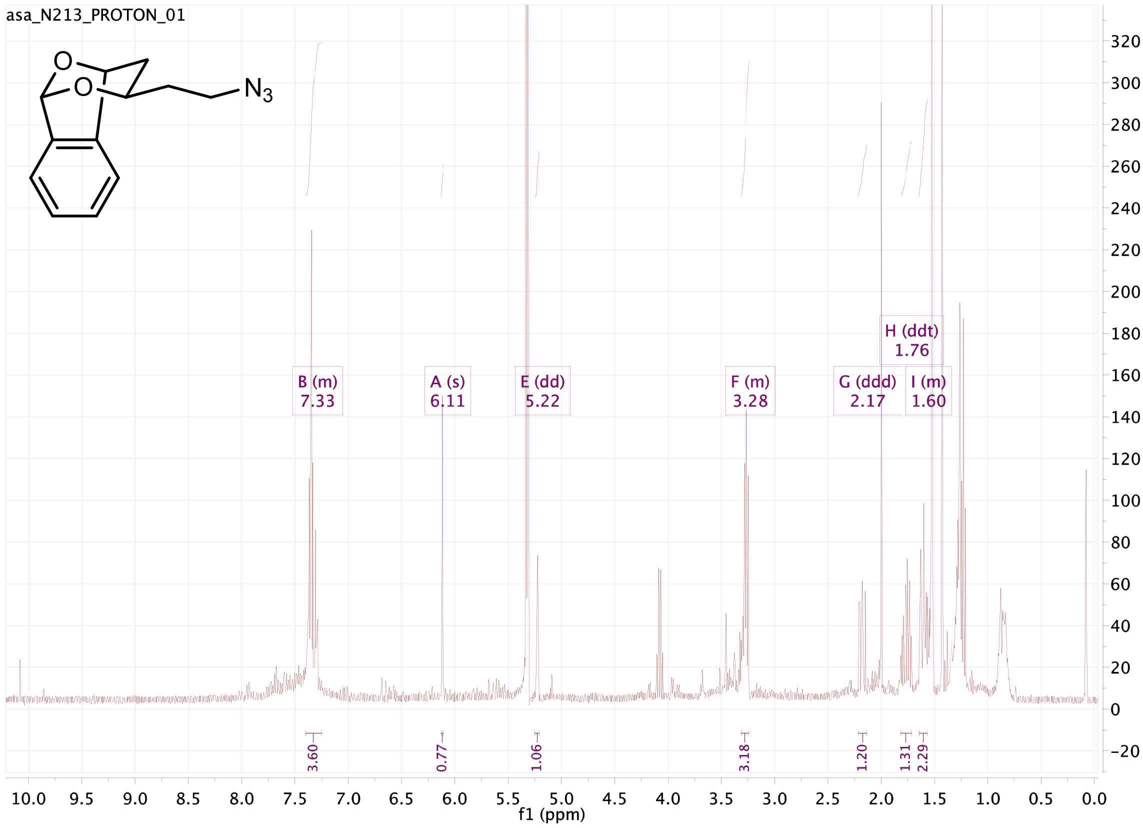

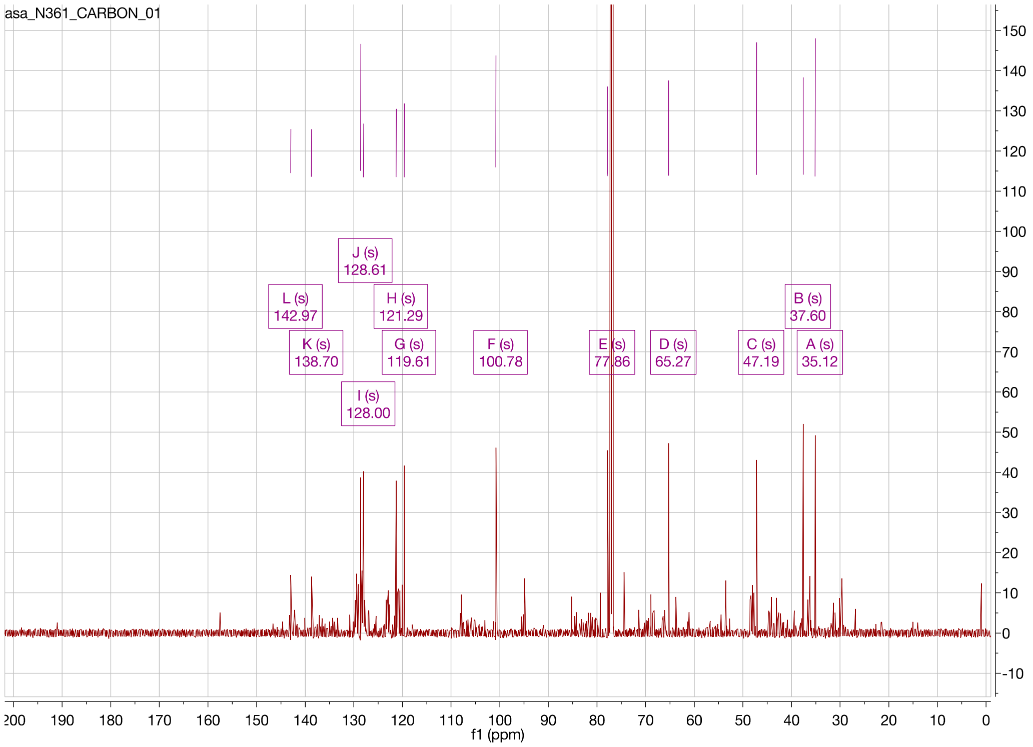

Figure S15. ^{1}H- (top) and ^{13}C-NMR spectrum (bottom) of **23a**.

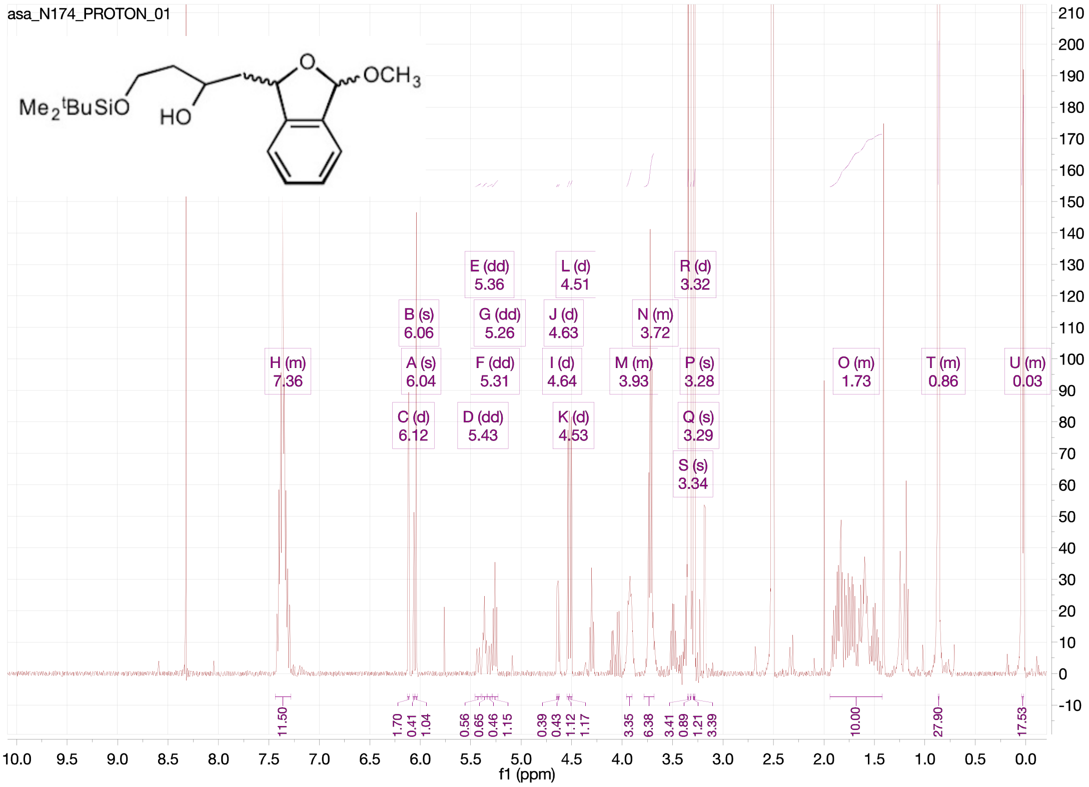

Figure S16. ^{1}H-NMR spectrum of **19a**.

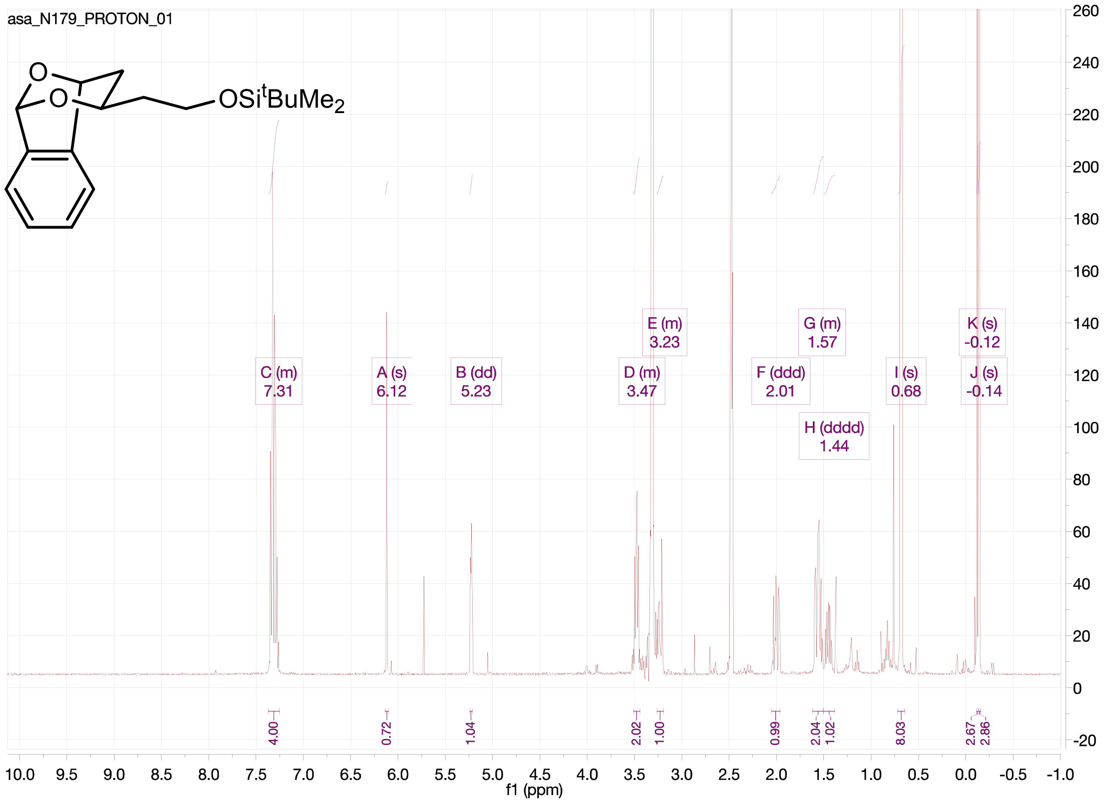

Figure S17. ^{1}H-NMR spectrum of **22a**.
